# Meta-Analysis on Prevalence and Attribution of Human Papillomavirus Types 52 and 58 in Cervical Neoplasia Worldwide

**DOI:** 10.1371/journal.pone.0107573

**Published:** 2014-09-17

**Authors:** Paul K. S. Chan, Wendy C. S. Ho, Martin C. W. Chan, Martin C. S. Wong, Apple C. M. Yeung, Josette S. Y. Chor, Mamie Hui

**Affiliations:** 1 Department of Microbiology, The Chinese University of Hong Kong, Prince of Wales Hospital, Shatin, New Territories, Hong Kong Special Administrative Region, People's Republic of China; 2 Jockey Club School of Public Health and Primary Care, Faculty of Medicine, The Chinese University of Hong Kong, Prince of Wales Hospital, Shatin, New Territories, Hong Kong Special Administrative Region, People's Republic of China; National Institute of Health - National Cancer Institute, United States of America

## Abstract

**Objective:**

To estimate the prevalence and attribution of two non-vaccine-covered HPV types (HPV52 and HPV58) across the world.

**Methods:**

Meta-analysis on studies reported in English and Chinese between 1994 and 2012.

**Results:**

The pooled prevalence and attribution rates of HPV52 and HPV58 in invasive cervical cancers were significantly higher in Eastern Asia compared to other regions (HPV52 prevalence: 5.7% vs. 1.8–3.6%, P<0.001; HPV52 attribution: 3.7% vs. 0.2–2.0%; HPV58 prevalence: 9.8% vs. 1.1–2.5%, P<0.001; HPV58 attribution: 6.4% vs. 0.7–2.2%, P<0.001). Oceania has an insufficient number of studies to ascertain the prevalence of HPV52. Within Eastern Asia, the attribution of HPV58 to invasive cervical cancer was 1.8-fold higher than that of HPV52. Similarly, HPV52 and HPV58 shared a higher prevalence and attribution among cervical intraepithelial neoplasia in Eastern Asia. In contrast to the classical high-risk type, HPV16, the prevalence and attribution of HPV52 and HPV58 decreased with increasing lesion severity. Thus, HPV52 and HPV58 behave as an “intermediate-risk” type.

**Conclusion:**

The attribution of HPV52 and HPV58 to cervical intraepithelial neoplasia and invasive cancer in Eastern Asia were respectively 2.5–2.8 and 3.7–4.9 folds higher than elsewhere. Changes in the attributed disease fraction can serve as a surrogate marker for cross-protection or type replacement following widespread use of HPV16/18-based vaccines. This unique epidemiology should be considered when designing HPV screening assays and vaccines for Eastern Asia.

## Introduction

Cervical cancer is the fourth most common cancer in women worldwide. There were about 528,000 new cases and 266,000 deaths reported in 2012 [1]. Persistent infection by certain types of human papillomaviruses (HPVs) is recognized as a necessary cause for cervical cancer [2]. There are more than 150 HPV types being identified and at least 15 of them are regarded as “high-risk” contributing to the development of cervical cancer [3]. HPV16 and HPV18 account for about 70% of invasive cervical cancers (ICC) worldwide, and the proportion is quite consistent throughout the world. HPV33, 45, 31, 58, 52 and 35 rank between the third and the eighth overall, but with more variation in the ranking of individual types across countries [4], [5]. Among women with high-grade intraepithelial neoplasia, HPV16 is still the most prevalent type found worldwide, but greater geographical variability has been observed for other HPV types [6]. For instance, HPV31 and HPV33 have been reported to rank the second and third in Africa and Europe, respectively; whereas HPV58 and HPV52 were the second and third most common types found in Asia, respectively [7].

In light of the geographical variation in prevalence and attribution of HPV52 and HPV58, and the fact that they have been found in a substantial proportion of ICC in Asia [5]–[15], we conducted a meta-analysis to assess the distribution of HPV52 and HPV58 among high-grade cervical intraepithelial neoplasia and invasive cancers worldwide.

## Materials and Methods

### Source of data

A systematic search on Medline and CNKI China Journals Full-text Database (http://big5.oversea.cnki.net/Kns55/brief/result.aspx?dbPrefix=CJFD) was performed to retrieve papers published from January 1994 to December 2012 on HPV. Combinations of the index terms: “HPV”, “HPV52”, “HPV58”, “epidemiology”, “cervical neoplasia” and “cervical cancer” were used for Medline; whereas “HPV”, “HPV52型”, “HPV58型”, “宮頸病變” and “宮頸癌” were used for CNKI database that records Chinese journals. Only papers published in English and Chinese were reviewed. We put a special effort to screen Chinese journals because of the reported high prevalence of HPV52 and HPV58 among Chinese populations [8]–[13]. Abstracts drawn based on these key words were screened. Those reports obviously not eligible for our study were eliminated. The remaining manuscripts were reviewed in detail against the inclusion criteria. Data including geographical source of sample, sample size, HPV (overall, HPV52/58) positive rates, HPV types found in multiple infections, testing method, histological diagnosis were recorded. Only information presented in the published manuscript was used. We did not contact the authors. The review process was done by the same experienced researcher all the way through.

### Inclusion criteria

Manuscripts were included in this meta-analysis only if they fulfilled all the following criteria: (1) were original study; (2) had examined at least 30 HPV-positive cases of histology-confirmed cervical intraepithelial neoplasia (CIN) or ICC, or at least 20 cases of adenocarcinoma and/or adenosquamous carcinoma; (3) had used PCR-based HPV detection methods that can detect HPV52 and/or HPV58; (4) had provided sufficient data to determine the relative prevalence of HPV52 and/or HPV58; (5) study subjects were not known to be infected with human immunodeficiency virus or otherwise immunocompromised. Repeated reports appeared to be derived from the same cohort were excluded. We set a minimum sample size as the inclusion criteria to minimize bias as we expected HPV52 and HPV58 to be uncommon in most studies.

### Data presentation and analysis

Continents and regions were grouped according to the classification of the United Nations (http://unstats.un.org/unsd/methods/m49/m49regin.htm). ICC included squamous cell carcinoma (SCC), adenocarcinoma and adenosquamous cell carcinoma (ADC), and those with unspecified histology (UNSPEC). Cervical squamous cell carcinoma or adenocarcinoma in-situ were grouped under CIN3. A vast majority of the UNSPEC cases included in this analysis were from studies that did not report histology at all. Since SCC predominates in ICC worldwide, SCC was grouped together with UNSPEC for the purpose of presenting prevalence and attribution across regions. Cases of “true UNSPEC” from studies that report histology were too few to form a separate category for analysis.

“Prevalence” refers to relative prevalence rate defined as the total number of HPV52/58-positive samples, regardless of the status of single-type or multiple-type infection, divided by the total number of HPV-positive samples for that disease category.

An approach published previously was used to approximate the attribution of HPV52/58 found in multiple-type infections [11], [16]. For samples harboring multiple HPV types, each type found was assumed to have a fractional attribution to the disease development, and this fraction would be proportional to the relative number of instances in which that HPV type was observed as single-type infection in that disease category. Briefly, an “attribution factor” for HPV52/58 was calculated based on the formula “number of samples with HPV52/58 single-type infection divided by the number of samples with single-type infection of any HPV type in that disease category”. The attribution of HPV52/58 for that disease category would be: (percentage of specimens with HPV52/58 alone) + (percentage of specimens with HPV52/58 co-exists with other HPV types multiplied by the attribution factor).

The 95% confidence intervals of prevalence and attribution rates were calculated by the Wilson procedure with a correction for continuity using an on line program available at http://www.vassarstats.net/prop1.html [17], [18]. Differences in pooled prevalence and attribution rates between regions were assessed by Chi-squared test. Two-sided P-values<0.05 were regarded as statistically significant.

## Results

**Figure 1:**
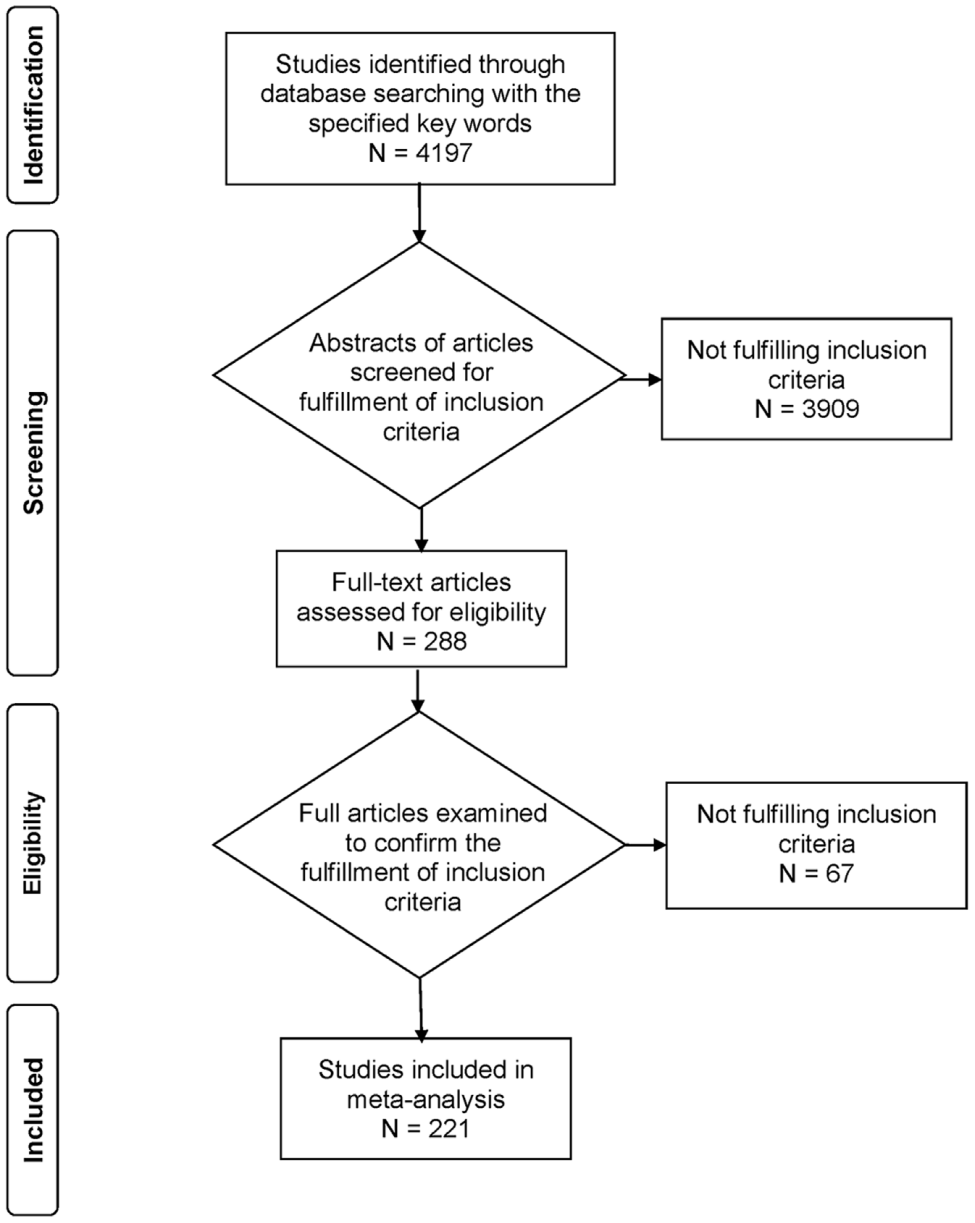
Study flow chart.

Altogether, 221 studies (152 in English and 69 in Chinese) fulfilled the inclusion criteria, and most of them (94.6%) provided data for both HPV52 and HPV58, whereas the rest only provided data for one type (2 studies for HPV52 and 10 studies for HPV58) (Figure 1). The majority of the studies were conducted in Asia (142, 64.3%), followed by Europe (42, 19.0%) and Americas (26, 11.8%). Only 12 studies were available from Africa and 8 from Oceania.

### Invasive cervical cancer (ICC) – squamous cell carcinoma and unspecified histology

#### HPV52 across continents

**Figure 2:**
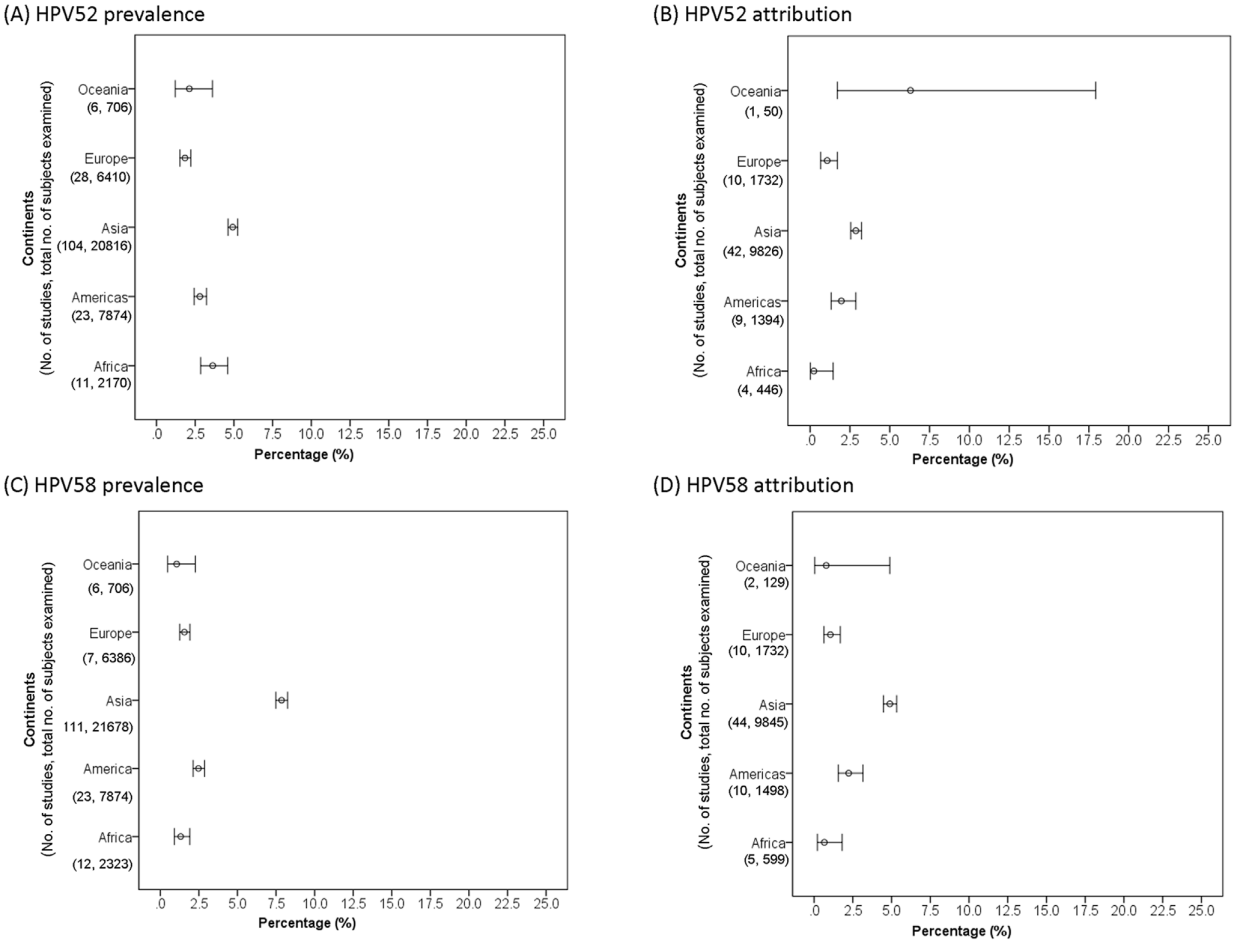
Cervical squamous cell carcinoma and invasive cervical cancer of unspecified histology across continents.

There were 162 studies available for analysis of HPV52 in ICC. Majority (76.7%) of these studies did not report histology data, and these cases were regarded as ICC of unspecified histology (most of these cases were expected to be squamous cell carcinoma) (Table S1). The prevalence of HPV52 among SCC and ICC of unspecified histology was significantly higher (P<0.01) in Asia (4.9%, 95% confidence interval 4.6–5.2) compared to Africa (3.6%, 2.9–4.6), Americas (2.8%, 2.4–3.2), Oceania (2.1%, 1.2–3.6) and Europe (1.8%, 1.5–2.2) (Figure 2A).

Sixty-six studies provided sufficient data for analysis on attribution of HPV52 to SCC and ICC of unspecified histology (Table S1). Asia showed the highest attribution rate (2.9%, 2.6–3.3), which was significantly higher (P<0.001) than Europe (1.1%, 0.7–1.7) and Africa (0.2%, 0.0–1.5), but not significantly different from Americas (2.0%, 1.3–2.9) (Fig 2B). Only one study from Oceania provided sufficient data for attribution analysis [19], and high prevalence (10.0%) and attribution (6.3%) rates were reported. Further studies are required to verify whether the finding is representative of Oceania.

#### HPV58 across continents

Altogether, 170 studies were available for analysis of HPV58 in ICC. Majority (75.6%) of these studies did not report histology data, and these cases were regarded as ICC of unspecified histology (most of these cases were expected to be squamous cell carcinoma) (Table S2). The prevalence of HPV58 reported from Asia (7.9%, 7.5–8.3%) was significantly (P<0.001) higher than Americas (2.5%, 2.1–2.9), Europe (1.6%, 1.3–2.0), Africa (1.3%, 0.9–1.9) and Oceania (1.1%, 0.5–2.3) (Figure 2C). Americas ranked second in HPV58 prevalence, which was significantly higher than those in Europe, Africa and Oceania (P = 0.03).

Asia also showed the highest attribution rate (4.9%, 4.5–5.3), which was significantly higher (P<0.001) than Americas (2.2%, 1.6–3.2), Europe (1.1%, 0.6–1.7%) and Africa (0.7%, 0.2–1.8) (Table S2) (Figure 2D). Only two studies with a total of 129 cases were available from Oceania resulting in a wide confidence interval for the pooled attribution rate [19], [20].

#### HPV52 within Asia

**Figure 3:**
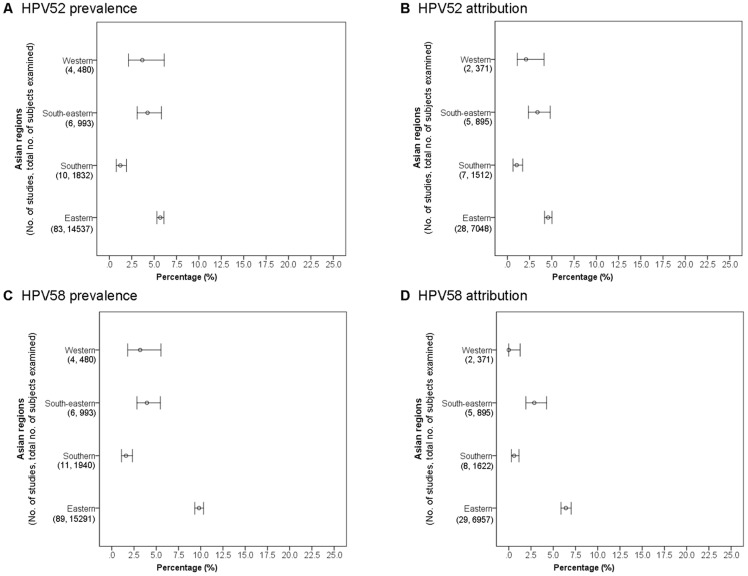
Cervical squamous cell carcinoma and invasive cervical cancer of unspecified histology across Asian regions.

There were 103 studies on SCC and/or ICC of unspecified histology available from Asia for analysis of HPV52 prevalence (Table S1). Eastern Asia including mainland China, Taiwan, Hong Kong, Macao, Japan and Korea showed the highest prevalence rate of 5.7% (5.3–6.1), but was not significantly different from South-eastern (4.3%, 3.1–5.8) and Western Asia (3.7%, 2.2–6.1) (Figure 3A). However, the number of cases from South-eastern and Western Asia was relative small, making a wide confidence interval. Southern Asia including India, Pakistan and Nepal showed a significantly lower (P<0.001) prevalence rate (1.2%, 0.8–1.9) compared to other Asian regions.

Eastern Asia showed the highest attribution rate (3.5%, 3.1–3.9) among SCC and ICC of unspecified histology, and was significantly higher (P<0.01) than those of Western (0.5%, 0.1–2.1) and Southern (0.9%, 0.5–1.5) Asia (Figure 3B).

#### HPV58 within Asia

Altogether, 110 studies from Asia were available for analysis of the prevalence of HPV58 in SCC and/or ICC of unspecified histology (Table S2). Eastern Asia showed a significantly (P<0.001) higher prevalence (9.8%, 9.3–10.3) compared to other Asian regions (1.6–4.0%) (Figure 3C).

Eastern Asia also showed a significantly (P<0.001) higher attribution (6.4%, 5.9–7.0) of HPV58 to SCC and ICC of unspecified histology compared to all other Asian regions (0–2.9%) (Table S2) (Figure 3D).

### Invasive cervical cancer (ICC) – adenocarcinoma and adenosquamous cell carcinoma

Cases of adenocarcinoma and adenosquamous cell carcinoma were pooled together and referred as ADC for the purpose of presenting prevalence and attribution rates. Altogether, 22 studies provided data for analysis of HPV52 and HPV58 prevalence in ADC (Tables S3 and S4).

#### HPV52/58 across continents

**Figure 4:**
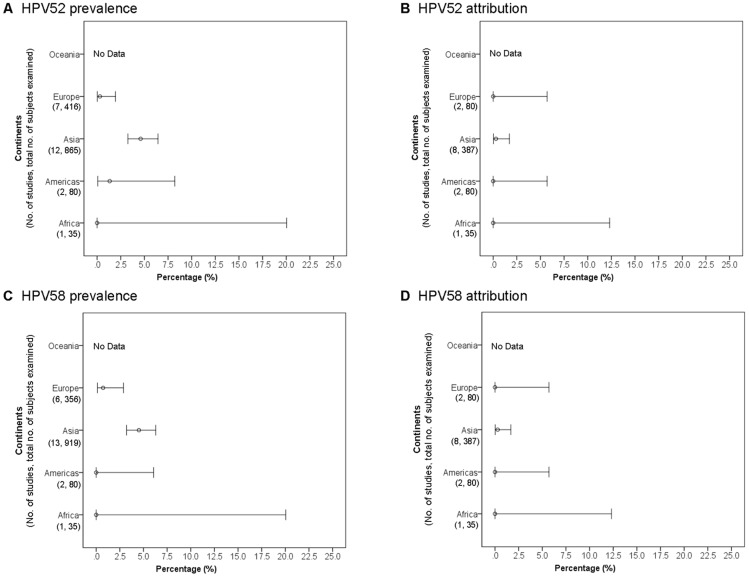
Cervical adenocarcinoma and adenosquamous cell carcinoma across continents.

Asia showed the highest prevalence rate for HPV52 (4.6%, 3.3–6.4), which was significantly higher than that of Europe (0.3%, 0.0–1.9) (Figure 4A). Only 13 studies provided sufficient data for attribution analysis resulting in a wide range of confidence intervals, and no significant difference between continents was observed (Figure 4B). Of note, the attribution of HPV52 to ADC in Asia was low, despite a relatively higher prevalence was observed, suggesting most were bystander co-infections.

The observations for HPV58 were similar to those of HPV52. The prevalence of HPV58 in ADC was highest in Asia (4.5%, 3.2–6.3), but only a low attribution rate was observed (0.3%, 0.0–1.7) (Figures 4C and 4D).

#### HPV52/58 within Asia

Data on ADC were only available from Eastern and South-Eastern Asia (Tables S3 and S4). The prevalence rates of HPV52 in ADC of these two Asian regions were similar (Eastern Asia: 4.6%, 3.2–6.6 vs. 4.3%, 1.4–11.3, P = 1.0), and no difference in attribution was observed (Eastern Asia: 0%, 0–1.7 vs. 1.1%, 0.1–6.4, P = 0.26).

### Cervical intraepithelial neoplasia grade 3

#### HPV52 across continents

**Figure 5:**
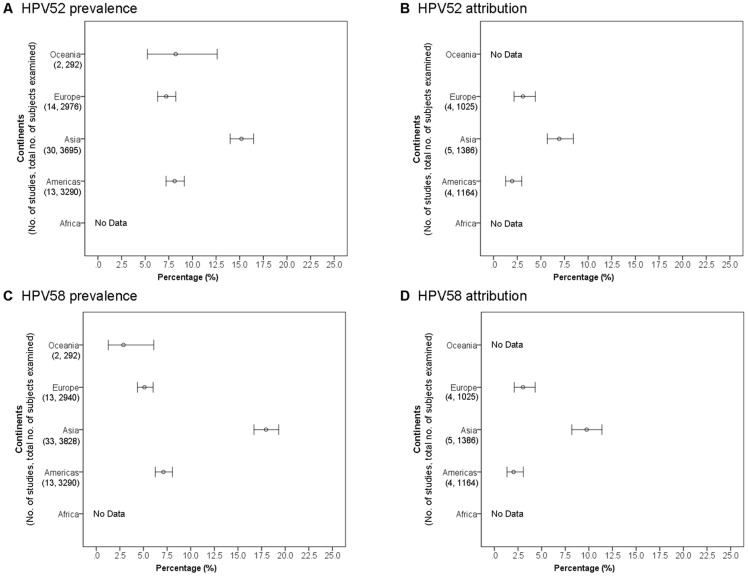
Cervical intraepithelial neoplasia grade 3 across continents.

Altogether, 59 studies were available for analysis (Table S5). The prevalence of HPV52 in CIN3 was highest in Asia (15.2%, 14.0–16.5), and was significantly higher than those of other regions (Americas: 8.1%, 7.2–9.1; Europe: 7.2%, 6.3–8.2; Oceania: 8.2%, 5.2–12.6; P≤0.01 for all others) (Figure 5A). No data was available from Africa.

The attribution of HPV52 to CIN3 was also found to be significantly higher in Asia (6.9%, 5.9–8.4) than in Americas (2.0%, 1.3–3.0, P<0.001) and Europe (3.1%, 2.2–4.4, P<0.001) (Figure 5B) (Table S5).

#### HPV58 across continents

Sixty-one studies were available (Table S6). The prevalence of HPV58 in CIN3 was significantly higher in Asia (18.0%, 16.7–19.3) than in Americas (7.1%, 6.3–8.1, P<0.001), Europe (5.1%, 4.4–6.0, P<0.001) and Oceania (2.9%, 1.3–6.1, P<0.001) (Figure 5C).

Similarly, the attribution of HPV58 to CIN3 was also significantly higher in Asia (8.8%, 8.2–11.4) than in Americas (2.2%, 1.4–3.2, P<0.001) and Europe (3.0%, 2.1–4.3, P<0.001) (Figure 5D).

### Cervical intraepithelial neoplasia grade 2

Altogether, 57 studies provided data for analysis on prevalence of HPV52 and HPV58 in CIN2 cases (Tables S7 and S8). Of note, only two studies were available from Oceania and none from Africa. Only 13 studies provide sufficient data for analysis on attribution.

#### HPV52 across continents

**Figure 6:**
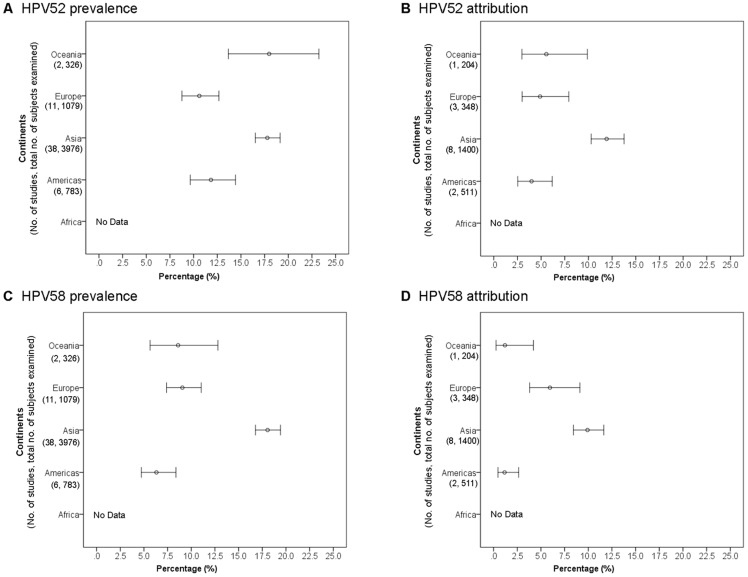
Cervical intraepithelial neoplasia grade 2 across continents.

The prevalence of HPV52 in CIN2 was significantly higher in Asia (17.8%, 16.5–19.1) than in Europe (10.6%, 8.8–12.7, P<0.001) and Americas (11.8%, 9.6–14.4, P<0.001) (Figure 6A). The prevalence of HPV52 in Oceania appeared to be high, but with a wide confidence interval. Of note, there were only two studies available from Oceania. Both were conducted in Melbourne by the same group reporting high prevalence rates of 19.6% and 14.1%, respectively [21], [22].

Similarly, the attribution of HPV52 to CIN2 in Asia (11.9%, 10.3–13.8) was significantly higher than Europe (4.9%, 3.0–7.9, P<0.001) and Americas (4.0%, 2.5–6.2, P<0.001) (Figure 6B).

#### HPV58 across continents

The prevalence of HPV58 among CIN2 cases in Asia (18.1%, 16.8–19.4) was significantly higher than in other regions (Europe: 9.1%, 7.4–11.1; Oceania: 8.6%, 5.7–12.8; Americas: 6.3%, 4.7–8.4; P<0.001) (Figure 6C).

The attribution of HPV58 to CIN2 in Asia (9.9%, 8.4–11.6) was significantly higher than in Americas (1.2%, 0.5–2.7, P<0.001), but not significantly different from that of Europe where a wide confidence interval was observed (6.0%, 3.8–9.1, P = 0.48) (Figure 6D).

### Different lesion grades within Eastern Asia

Further analyses were focused on Eastern Asia where HPV52 and HPV58 showed the highest disease burden. The attribution rates of HPV52 to CIN2/3 and ICC were 2.5- to 2.8-fold higher for cases in Eastern Asia compared to other parts of the world, whereas the corresponding attribution rates for HPV58 were 3.7- to 4.9-fold higher in Eastern Asia (Table 1).

**Table 1:** Prevalence and attribution of HPV52 and HPV58 across different lesion grades in Eastern Asia and other parts of the world.

Cervical lesion grade	Eastern Asia (prevalence/attribution rate in%, 95% confidence interval)	Other regions (prevalence/attribution rate in%, 95% confidence interval)
HPV52 prevalence^1^		
CIN2	17.8, 16.5–19.1	12.0, 10.6–13.5
CIN3	15.2, 14.0–16.5	7.7, 7.1–8.4
Adenocarcinoma	4.6, 3.2–6.6	1.2, 0.5–2.6
SCC/UNSPEC	5.7, 5.3–6.1	2.7, 2.5–2.9
HPV52 attribution^2^		
CIN2	11.9, 10.3–13.8	4.6, 3.5–6.1
CIN3	6.9, 5.7–8.4	2.5, 1.9–3.3
Adenocarcinoma	0.0, 0.0–1.7	0.4, 0.0–2.2
SCC/UNSPEC	3.5, 3.1–3.9	1.4, 1.1–1.7
HPV58 prevalence^3^		
CIN2	18.1, 16.8–19.4	8.0, 6.9–9.3
CIN3	18.0, 16.7–19.3	6.0, 5.5–6.7
Adenocarcinoma	5.0, 3.5–7.0	0.6, 0.2–2.1
SCC/UNSPEC	9.8, 9.3–10.3	2.0, 1.8–2.2
HPV58 attribution^4^		
CIN2	9.9, 8.4–11.6	2.7, 1.9–3.9
CIN3	9.7, 8.2–11.4	2.5, 1.9–3.3
Adenocarcinoma	0.4, 0.0–2.3	0.0, 0.0–1.6
SCC/UNSPEC	6.4, 5.9–7.0	1.3, 1.1–1.7

1 Relative prevalence, no. of HPV52-positive cases regardless of single- or multiple-type infection/total no. of HPV-positive cases.

2 % of cases with HPV52 single-type infection +% of cases with HPV52 multiple-type infection × attribution factor. Attribution factor  =  no. of cases with HPV52 single-type infection/no. of cases with single-type infection of any HPV type.

3 Relative prevalence, no. of HPV58-positive cases regardless of single- or multiple-type infection/total no. of HPV-positive cases.

4 % of cases with HPV58 single-type infection +% of cases with HPV58 multiple-type infection × attribution factor. Attribution factor  =  no. of cases with HPV58 single-type infection/no. of cases with single-type infection of any HPV type.

CIN, cervical intraepithelial neoplasia; SCC/UNSPEC, squamous cell carcinomas and invasive cervical cancers of unspecified histology; adenocarcinoma includes cervical adenocarcinoma and adenosquamous cell carcinoma.

**Figure 7:**
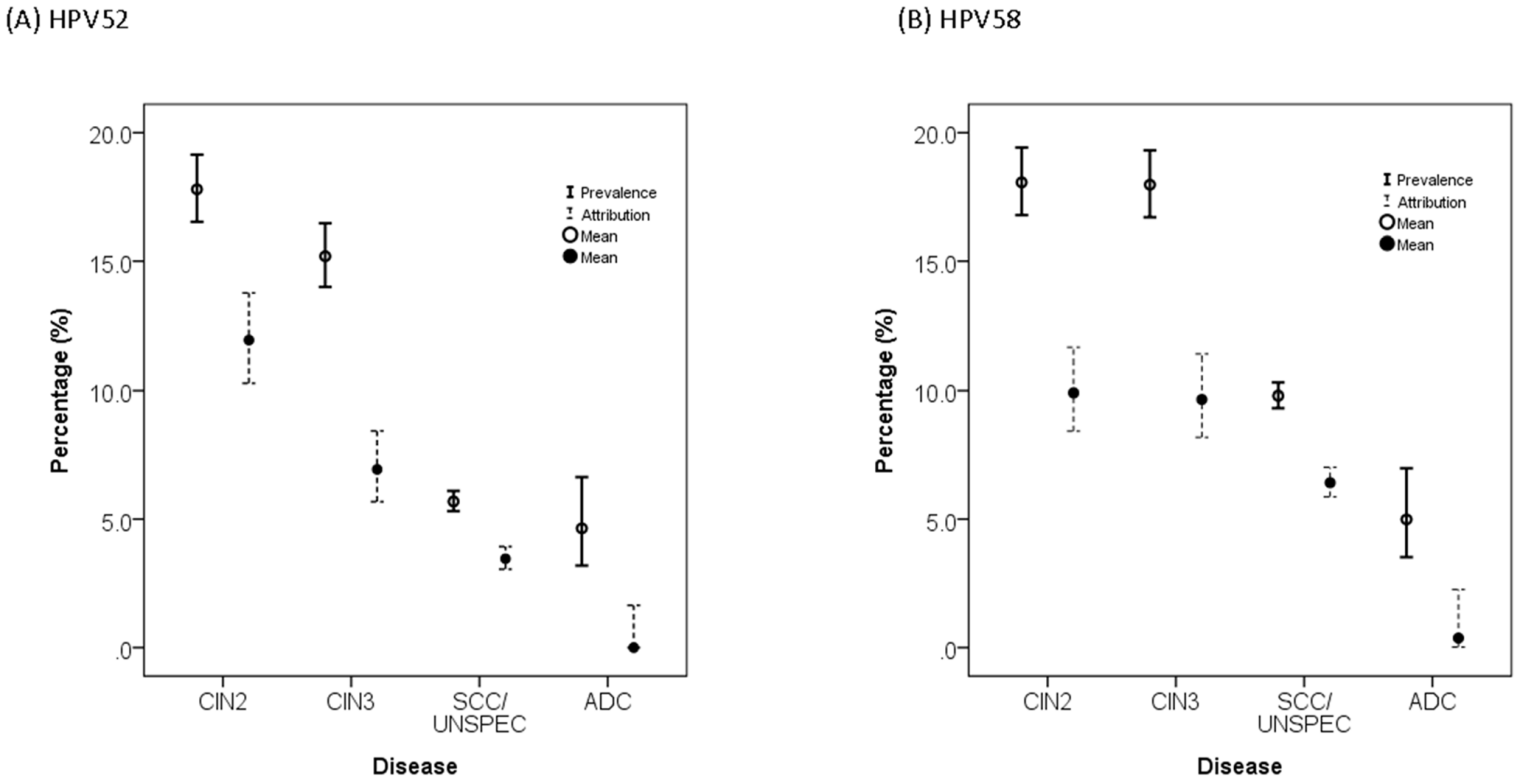
Prevalence and attribution of HPV52 and HPV58 among cervical lesions in Eastern Asia.

The prevalence and attribution rates of both HPV52 and HPV58 showed a significant linear trend of decrease with an increase in lesion severity from CIN2 to ICC (Chi-squared test for linear trend: 29 to 593, P<0.001) (Figure 7A, 7B). The ratio of prevalence/attribution rate, which reflects the proportion of co-infection, dropped from CIN3 to invasive cancer for both HPV52 and HPV58 (HPV52: from 2.2 to 1.6, HPV58: from 1.9 to 1.5).

## Discussion

The eventual decrease in incidence of cervical cancer will be an ultimate proof of success of prophylactic HPV vaccines. In addition to the change in absolute number of cases, any shift in the proportion of cancers associated with vaccine-covered and non-vaccine-covered types is an important evidence of cross-type protection and type replacement. Such shift would be easier to observe in countries where non-vaccine-covered types share a larger proportion of cases.

The current meta-analysis consolidates previous observations that the prevalence of HPV52 and HPV58 was higher, across all lesion grades ranging from CIN2 to ICC, in Asia compared to all other continents. We choose to use relative prevalence (i.e. positive rate among HPV-positive samples) rather than crude prevalence to minimize bias due to variation in analytical sensitivity across studies. However, one should note that there could be residual bias because of different sensitivities of techniques for specific HPV types, and other sources of potential variation, e.g. type of tissue preservation, that we did not adjust.

To ascertain the role of HPV52/58 found in multiple infections, we adopted an approach used in previous studies to estimate the attribution rate [11], [16]. The advantage of using “attribution” is to avoid over-estimating the contribution of a certain HPV type in case of multiple infections. However, using “attribution” may potentially have a bias towards studies having less multiple infections, thus it is easier for them to present the different combinations in the report. Of note, a large proportion of the available studies only reported the overall prevalence of individual HPV types, and did not differentiate them into single-type and multiple-type infection, rendering the calculation of attribution rate not possible. Further studies should employ methods that can identify multiple HPV types simultaneously, and data should be presented according to the status of single-type and multiple-type infection. Using the “attribution” approach, we confirmed that for all lesion grades ranging from CIN2 to ICC, except adenocarcinoma/adenosquamous cell carcinoma, HPV52/58 attributed to a highest proportion of cases in Asia compared to other continents.

Australia was one of the few countries adopting national immunization program soon after the approval of prophylactic HPV vaccines, and is going to be one of the first few countries to demonstrate the benefit of HPV vaccination at a population level. However, only limited data on HPV52/58 were available from Australia and most reports were not detailed enough to assess attribution. Based on the two available studies, a high prevalence of HPV52 in CIN2 was observed in Melbourne [21], [22]. More studies from Australia would help clarifying the role of HPV52, as well as assessing any cross-protection or type replacement in relation to wide-spread use of HPV16/18-based vaccines.

Further analysis on studies conducted in Asia showed that the high prevalence and attribution of HPV52 and HPV58 in cervical squamous cell carcinoma and ICC of unspecified histology was contributed by Eastern Asia where the data were mainly derived from genetically related populations including Chinese, Japanese and Korean. In a recent meta-analysis reported by Guan et al., the prevalence of HPV52 among invasive cervical cancers in Eastern Asia was estimated to be 6.1%, which was about 2- to 4-fold higher than other regions; whereas the corresponding prevalence of HPV58 was 10.2%, which was 4- to 12-folds higher than other regions. [23] Guan et al. also reported a much higher prevalence of HPV52 and HPV58 among low-grade and high-grade lesions in Eastern Asia compared to other regions. These findings are in line with our observations, and we further confirmed the impact of HPV52 and HPV58 by analyzing their attribution rates in different degrees of cervical lesion. While the underlying reasons for the higher disease impact of HPV52 and HPV58 in East Asia could be multiple, previous studies on southern Chinese have shown a positive risk association between HLA II DQB1*06 and HPV58-postive CIN3/ICC [24], and a protective effect of HLA-B15 for HPV52-positive CIN3/ICC [25], suggesting genetic background may play a role in susceptibility to development of cervical neoplasia and the effect could be HPV type-specific. Furthermore, it has reported that Asia is dominated by different lineages of HPV52 and HPV58 variants. [26], [27] It is worthwhile to investigate whether skewed geographical distribution of variants is linked with the pattern of disease impact observed across the globe.

While the current HPV16/18-based prophylactic vaccines confer a limited cross-protection against additional types including HPV52 and HPV58 [28]–[31], the efficacy is likely not high enough. Surveillance on the prevalence and attribution of HPV52 and HPV58 following wide spread administration of vaccines in East Asia is important to identify type replacement early if it ever happens.

Our findings indicate that surveillance for the prevalence and attribution of HPV52 and HPV58 in East Asia following wide spread administration of HPV16/18-based prophylactic vaccines is important. While both HPV52 and HPV58 are more prevalent in Asia, especially Eastern Asia, HPV58 plays a more important role. We found that the attribution of HPV58 to cervical cancer was 1.8 higher than that of HPV52. In addition to host genetic background, oncogenic risk of HPV variant may play a role. It has been shown that an HPV58 variant carrying E7 mutations T20I and G63S was associated with a 6.9-fold increase in risk for cervical cancer compared to HPV58 variants without these amino acid substitutions [32]. In another study on the distribution of HPV58 variants across the world, these high-risk substitutions were found in 33% of HPV58 isolates in Asia, 10% in Americas, 3% in Europe and none in Africa [33]. The risk association and geographical distribution of this “high-risk HPV58 variant” corroborate with the observation of this meta-analysis.

Based on the model of HPV16, one would expect the prevalence of an high-risk type to increase with the severity of lesion [11], [16], [34]. In contrast, a trend of decrease in prevalence and attribution was observed for HPV52 and HPV58, suggesting that most of the intraepithelial lesions, even in Eastern Asia, would be cleared by itself. This pattern of decrease in prevalence from intraepithelial neoplasia to invasive cancer was also observed in the meta-analysis reported by Guan et al. [23]. In this regard, HPV52 and HPV58 are less oncogenic than the classical high-risk types HPV16 and HPV18.

In conclusion, HPV52 and HPV58 account for a higher proportion of cervical intraepithelial neoplasia and invasive cancers in Eastern Asia compared to other parts of the world. Changes in the attributed disease fraction can serve as a surrogate mark for cross-protection or type replacement following widespread use of HPV16/18-based vaccines. This unique epidemiology of cervical HPV infection in Eastern Asia should be considered when designing HPV screening assays and vaccines.

## Supporting Information

Table S1 **Ranking, relative prevalence and attribution of HPV52 among squamous cell carcinoma and invasive cervical cancer of unspecified histology reported from 162 studies.**
(DOCX)

Table S2 **Ranking, relative prevalence and attribution of HPV58 among squamous cell carcinoma and invasive cervical cancer of unspecified histology reported from 170 studies.**
(DOCX)

Table S3 **Ranking, relative prevalence and attribution of HPV52 among cervical adenocarcinoma and adenosquamous cell carcinoma reported from 22 studies.**
(DOCX)

Table S4 **Ranking, relative prevalence and attribution of HPV58 among cervical adenocarcinoma and adenosquamous cell carcinoma reported from 22 studies.**
(DOCX)

Table S5 **Ranking, relative prevalence and attribution of HPV52 among cervical intraepithelial neoplasia grade 3 reported from 59 studies.**
(DOCX)

Table S6 **Ranking, relative prevalence and attribution of HPV58 among cervical intraepithelial neoplasia grade 3 reported from 61 studies.**
(DOCX)

Table S7 **Ranking, relative prevalence and attribution of HPV52 among cervical intraepithelial neoplasia grade 2 reported from 57 studies.**
(DOCX)

Table S8 **Ranking, relative prevalence and attribution of HPV58 among cervical intraepithelial neoplasia grade 2 reported from 57 studies.**
(DOCX)

Checklist S1 **PRISMA checklist.**
(DOC)
