# Supplementary material for: Meta-Analysis on Prevalence and Attribution of Human Papillomavirus Types 52 and 58 in Cervical Neoplasia Worldwide
Source: PLoS One. 2014 Sep 17;9(9):e107573. doi: 10.1371/journal.pone.0107573 (PMC4168000; doi:10.1371/journal.pone.0107573)
Supplement: Table S2 — Ranking, relative prevalence and attribution of HPV58 among squamous cell carcinoma and invasive cervical cancer of unspecified histology reported from 170 studies. (DOCX) [file pone.0107573.s002.docx]

**Table S2. Ranking, relative prevalence and attribution of HPV58 among squamous cell carcinoma and invasive cervical cancer of unspecified histology reported from 170 studies.**

| **Continent** | **Region** | **City/Country** | **Study period** | **HPV typing method** | **No of cases examined** | **No. of HPV- positive cases** | **HPV positive rate** | **Ranking of HPV58** | **No of HPV58- positive cases** | **Relative prevalence of HPV58^1^** | **Attribution^2^ of HPV58** | **Reference** | **Reporting language** |
| --- | --- | --- | --- | --- | --- | --- | --- | --- | --- | --- | --- | --- | --- |
| Africa | Eastern Africa | Ethiopia | 2000-2004 | Cloning and sequencing | 153 | 152 | 99.3% | 3^rd^ | 1 | 0.7% | 0.7% | Fanta BE, et al. Ethiop Med J. 2005;43:151-8. | English |
| Africa | Eastern Africa | Uganda | 1968-1992 | HPV SPF10-LiPA25 | 146 | 92 | 63.0% | NA | 0 | 0.0% | 0.0% | Odida M, et al. BMC Infect dis. 2008;8:85. | English |
| Africa | Eastern Africa | Mozambique | 2002-2006 | Luminex (Multimetrix, Germany) and sequencing | 221 | 200 | 90.5% | 8th | 5 | 2.3% | -- | Naucler P, et al. J Gen Virol. 2011;92:2784-91. | English |
| Africa | Eastern Africa | Mozambique | 2008^3^ | HPV SPF10-LiPA25 | 230 | 225 | 97.8% | 9th | 4 | 1.8% | -- | Castellsague X, et al. Int J Cancer. 2008;122:1901-4. | English |
| Africa | Eastern Africa | Mozambique | 2002-2003 | Dot-blot hybridization | 72 | 70 | 97.2% | 8th | 1 | 1.4% | 1.4% | Naucler P, et al. J Gen Virol. 2004;85:2189-90. | English |
| Africa | Eastern Africa | Zimbabwe | 1999-2002 | Restriction fragment length polymorphism | 98 | 95 | 96.9% | 5th | 1 | 1.1% | 1.0% | Stanczuk GA, et al. Acta Obstet Gynecol Scand. 2003;82:762-6. | English |
| Africa | Northern Africa | Tunisia | 2009-2010 | Luminex (Multimetrix, Germany) and dot-blot hybridization | 130 | 117 | 90.0% | 6th | 2 | 1.7% | 0.7% | KrennHrubec K, J Med Virol. 2011;83:651-7. | English |
| Africa | Northern Africa | Algeria | 1997-1999 | Type-specific PCR | NA | 156 | NA | 8th | 1 | 0.6% | -- | Hammouda D, et al. Int J Cancer. 2005;113:483-9. | English |
| Africa | Southern Africa | South Africa | 1993-1997 | Restriction fragment length polymorphism | 50 | 47 | 94.0% | 3rd | 1 | 2.1% | -- | Kay P, et al. J Med Virol. 2003;71:265-73 | English |
| Africa | Southern Africa | South Africa | 2002^3^ | Sequencing | 190 | 187 | 98.4% | 6th | 2 | 1.1% | -- | Pegoraro RJ, et al. Int J Gynecol Cancer. 2002;12:383-8 | English |
| Africa | Across sub-regions | 6 countries | 1989-1992 | PCR and 26HR probes | 186 | 167 | 89.8% | 5th | 5 | 3.0% | -- | Bosch FX, et al. J Natl Cancer Inst. 1995;87:796-802 | English |
| Africa | Across sub-regions | 4 countries | 1949-2009 | HPV SPF10-LiPA25 | 691 | 544 | 78.7% | 9th | 4 | 0.7% | -- | de Sanjose S, et al. Lancet Oncol. 2010;11:1048-56 | English |
| Americas | Latin America and the Caribbean (Central America) | Costa Rica | 1993-1994 | Dot-blot hybridization | 35 | 34 | 97.1% | 3rd | 4 | 11.8% | -- | Herrero R, et al. J Infect Dis. 2005;191:1796-807. | English |
| Americas | Latin America and the Caribbean (Central America) | Honduras | 1993-1995 | Sequencing | 104 | 83 | 79.8% | 4th | 7 | 8.3% | 6.7% | Ferrera A, et al. Int J Cancer. 1999;82:799-803. | English |
| Americas | Latin America and the Caribbean (Central America) | Mexico | 1998^3^ | Dot-blot hybridization | 69 | 60 | 87.0% | 5th | 3 | 5.0% | -- | Torroella-Kouri M, et al. Gynecol Oncol. 1998;70:115-20. | English |
| Americas | Latin America and the Caribbean (Central America) | Mexico | 1997-2003 | Restriction fragment length polymorphism | 119 | 119 | 100.0% | 5th | 5 | 4.2% | -- | Illades-Aguiar B, et al. Cancer Detect Prev. 2009;32:300-7. | English |
| Americas | Latin America and the Caribbean (Central America) | Mexico, Guerrero | 1998-2006 | Restriction fragment length polymorphism | 141 | 141 | 100.0% | 5th | 5 | 3.5% | -- | Illades-Aguiar B, et al. Gynecol Oncol. 2010;117:291-6. | English |
| Americas | Latin America and the Caribbean (Central America) | Mexico | 2006^3^ | Sequencing | 108 | 99 | 91.7% | 4th | 2 | 2.0% | -- | Pina-sanchez P, et al. Int J Gynecol Cancer. 2006;16:1041-7. | English |
| Americas | Latin America and the Caribbean (South America) | Brazil | 2001-2002 | Dot-blot hybridization | 48 | 41 | 85.4% | 2nd | 6 | 14.6% | 8.80% | Fernandes JV, et al. Mol Med Rep. 2011;4:1321-6. | English |
| Americas | Latin America and the Caribbean (South America) | Brazil | 1996-2000 | Dot-blot hybridization | 74 | 68 | 91.9% | 2nd | 8 | 11.8% | 9.6% | Fernandes JV, et al. BMC Res Notes. 2010;3:96. | English |
| Americas | Latin America and the Caribbean (South America) | Brazil | 2000^3^ | Restriction fragment length polymorphism | 59 | 53 | 89.8% | 4th | 2 | 3.8% | -- | Lorenzato F, et al. Int J Gynecol Cancer. 2000;10:143-150. | English |
| Americas | Latin America and the Caribbean (South America) | Paraguay | 1988-1990 | Hybridization | 113 | 109 | 96.5% | 6th | 4 | 3.7% | 2.7% | Rolon PA, et al. Int J Cancer. 2000;85:486-91. | English |
| Americas | Latin America and the Caribbean (South America) | Venezuela | 2001-2011 | INNO-LiPA | 96 | 95 | 99.0% | 4th | 3 | 3.2% | 3.1% | Sanchez-Lander J, et al. Cancer epidemiol. 2012;36:e284-7. | English |
| Americas | Latin America and the Caribbean (South America) | Chile | 2002-2005 | Reverse-line blot hybridization | 293 | 276 | 94.2% | 7th | 7 | 2.5% | 1.0% | Roa JC, et al. Int J Gynecol Obstet. 2009;105:150-3. | English |
| Americas | Latin America and the Caribbean (South America) | Peru | 1996-1997 | Southern blot hybridization | 171 | 163 | 95.3% | 7th | 4 | 2.5% | 1.2% | Santos C, et al. Br J Cancer. 2001;85:966-71. | English |
| Americas | Latin America and the Caribbean (South America) | Paraguay | 1960-2004 | HPV SPF10-LiPA25 | 432 | 316 | 73.1% | 8th | 3 | 1.0% | 0.5% | Kasamatsu E, et al. J Med Virol. 2012;84:1628-35. | English |
| Americas | Northern America | United States of America, New York | 1992-2007 | INNO-LiPA | 60 | 60 | 100.0% | 4th | 2 | 3.3% | 1.7% | Quint KD, et al. Gynecol Oncol. 2010;117:297-301. | English |
| Americas | Northern America | United States of America, New Mexico | 1980-1999 | Reverse-line blot hybridization | 808 | 735 | 91.0% | 8th | 20 | 2.7% | -- | Wheeler CM, et al. J Natl Cancer Inst. 2009;101:475-87. | English |
| Americas | Northern America | United States of America | 1949-2009 | HPV SPF10-LiPA25 | 176 | 160 | 90.9% | 5th | 3 | 1.9% | -- | de Sanjose S, et al. Lancet Oncol. 2010;11:1048-56. | English |
| Americas | Northern America | United States of America, Oklahoma | 2003-2007 | Linear array HPV genotyping (Roche) | 107 | 97 | 90.7% | 9th | 1 | 1.0% | 0.9% | Wentzensen N, et al. Int J Cancer. 2009;125:2151-8. | English |
| Americas | Northern America | United States of America, Oklahoma | 2007^3^ | Linear array HPV genotyping (Roche) | 93 | 87 | 93.5% | NA | 0 | 0.0% | -- | Zuna RE, et al. Mod Pathol. 2007;20:167-74. | English |
| Americas | Northern America | United States of America and Canada | 1989-1992 | PCR and 26HR probes | 57 | 53 | 93.0% | NA | 0 | 0.0% | -- | Bosch FX, et al. J Natl Cancer Inst. 1995;87:796-802. | English |
| Americas | Northern America | United States of America, California | 2007-2008 | Dot-blot hybridization | NA | 35 | NA | NA | 0 | 0.0% | -- | Castle PE, et al. Cancer Epidemiol Biomarkers Prev. 2011;20:946-53. | English |
| Americas | Across sub-regions | 8 countries | 1989-1992 | PCR and 26HR probes | 505 | 469 | 92.9% | 9th | 11 | 2.3% | -- | Bosch FX, et al. J Natl Cancer Inst. 1995;87:796-802. | English |
| Americas | Across sub-regions | 10 countries | 1949-2009 | HPV SPF10-LiPA25 | 4171 | 3404 | 81.6% | 9th | 67 | 2.0% | -- | de Sanjose S, et al. Lancet Oncol. 2010;11:1048-56 | English |
| Asia | Eastern Asia | China, Zhejiang | 2010-2011 | Hybridization | 50 | 48 | 96.0% | 1st | 28 | 58.3% | -- | Fang M, et al. Chin J Nosocomiol. 2012;22:685-7. | Chinese |
| Asia | Eastern Asia | China, Wenling | 2006-2008 | Type-specific PCR | 78 | 71 | 91.0% | 3rd | 23 | 32.4% | -- | Yang H, et al. Chin J Birth Health & Heredity. 2010;18:42-3. | Chinese |
| Asia | Eastern Asia | China, Shanghai | 1997^3^ | Hybridization and sequencing | 35 | 31 | 88.6% | 3rd | 9 | 29.0% | 14.8% | Huang S, et al. Int J Cancer. 1997;70:408-11. | English |
| Asia | Eastern Asia | China, Sichuan | 2006 | Type-specific PCR | 72 | 70 | 97.2% | 2nd | 19 | 27.1% | 8.2% | Wang AR, et al. Mod Prev Med. 2008;35:807-12. | Chinese |
| Asia | Eastern Asia | Taiwan | 1992-1996 | Restriction Fragment Length Polymorphism | 94 | 81 | 86.2% | 2nd | 17 | 21.0% | -- | Lai HC, et al. Int J Cancer. 1999;84:553-7. | English |
| Asia | Eastern Asia | China, Sichuan | 2004-2006 | Type-specific PCR and sequencing | 190 | 177 | 93.2% | 2nd | 37 | 20.9% | 3.5% | Wu EQ, et al. BMC Cancer. 2008;8:202. | English |
| Asia | Eastern Asia | China, Gansu | 2008-2010 | Microarray | 216 | 194 | 89.8% | 2nd | 40 | 20.6% | -- | Wang F, et al. Maternal and Child Health Care of China. 2011;26:2339-41. | Chinese |
| Asia | Eastern Asia | Republic of Korea | 1999^3^ | Restriction fragment length polymorphism and type-specific PCR | 38 | 36 | 94.7% | 3rd | 7 | 19.4% | 16.3% | Hwang T. J Korean Med Sci. 1999;14:593-9. | English |
| Asia | Eastern Asia | China, Sichuan | 2003-2004 | Type-specific PCR | 153 | 153 | 100.0% | 2nd | 29 | 19.4% | 11.2% | Qiu AD, et al. Gynecol Oncol. 2007;104:77-85. | English |
| Asia | Eastern Asia | China, Guizhou | 2007-2011 | NA | 53 | 44 | 83.0% | 1st | 8 | 18.2% | -- | Zhao S, et al. Maternal and Child Health Care of China. 2012;27:1632-5. | Chinese |
| Asia | Eastern Asia | China, Shanxi | 2001-2003 | Type-specific real-time PCR | 65 | 57 | 87.7% | 3rd | 10 | 17.5% | -- | Gao YE, et al. Sheng Wu Hua Xue Yu Sheng Wu Wu Li Xue Bao. 2003;35:1029-34. | English |
| Asia | Eastern Asia | China, Guizhou | 2006-2010 | HybriMax (HybriBio Limited, China) | 145 | 52 | 35.9% | 1st | 9 | 17.3% | -- | Sun L, et al. Guizhou Med J. 2011;35:883-7. | Chinese |
| Asia | Eastern Asia | Taiwan | 1993-2000 | Easychip HPV genotyping array (King Car, Taiwan) and Type-specific PCR | 2118 | 2046 | 96.6% | 3rd | 346 | 16.9% | 10.4% | Lai CH, et al. Int J Cancer. 2007;120:1999-2006. | English |
| Asia | Eastern Asia | China, Zhejiang | 2009-2011 | Hybrimax (HybriBio Limited, China) | 68 | 65 | 95.6% | 2nd | 11 | 16.9% | -- | Rao H, et al. Chin J Health Lab Tech. 2012;22:121-2. | Chinese |
| Asia | Eastern Asia | China, Shandong | 2008-2009 | Hybrimax (HybriBio Limited, China) | NA | 31 | NA | 3rd | 5 | 16.1% | -- | Li J, et al. Maternal and Child Health Care of China. 2012;27:195-8. | Chinese |
| Asia | Eastern Asia | China, Jiangsu | 2011 | Hybrimax (HybriBio Limited, China) | 32 | 31 | 96.9% | 3rd | 5 | 16.1% | -- | Wang Q, et al. Int J Lab Med. 2012;33:2019-21. | Chinese |
| Asia | Eastern Asia | China, Zhejiang | 2006-2009 | Hybridization | 40 | 38 | 95.0% | 3rd | 6 | 15.8% | -- | Zhou YQ, et al. Chin J Health Lab Tech. 2010;20:2868-70. | Chinese |
| Asia | Eastern Asia | China, Xinjing | 2009-2011 | Hybridization | 80 | 73 | 91.3% | 3rd | 11 | 15.1% | -- | Zhang X, et al. J Chin Prac Diag & Therapy. 2012;26:321-3. | Chinese |
| Asia | Eastern Asia | China, Zhejiang | 2008-2011 | Hybridization | 432 | 432 | 100.0% | 2nd | 63 | 14.6% | -- | Shou J. Chin J Nosocomiol. 2012;22:1336-7. | Chinese |
| Asia | Eastern Asia | China, Zhengzhou | 2006-2007 | HybriMax (HybriBio Limited, China) | 75 | 70 | 93.3% | 2nd | 10 | 14.3% | -- | Liu HY, et al. J Clin Exp Pathol. 2009;25:119-22. | Chinese |
| Asia | Eastern Asia | Taiwan | 2006^3^ | Easychip HPV genotyping array (King Car, Taiwan) | 81 | 71 | 87.7% | 4th | 10 | 14.1% | -- | Ho CM, et al. Gynecol Oncol. 2006;102:54-60. | English |
| Asia | Eastern Asia | Japan | 1999-2004 | HPV DNA Array | 60 | 59 | 98.3% | 5th | 8 | 13.6% | -- | Watari H, et al. pathobiology. 2011;78:220-6. | English |
| Asia | Eastern Asia | China, Kansu | 2006-2009 | INNO-LiPA | 48 | 46 | 95.8% | 3rd | 6 | 13.0% | -- | Lau K. Maternal and Child Health Care of China. 2010;25:2208-9. | Chinese |
| Asia | Eastern Asia | China, Hong Kong Special Administration Region | 2012^3^ | Linear array HPV genotyping (Roche) | 339 | 325 | 95.9% | 4th | 41 | 12.60% | 5.50% | Chan PK, et al. Int J Cancer. 2011;131:692-705. | English |
| Asia | Eastern Asia | China, Shenzhen | 2009^3^ | HPV-DNA chip | 40 | 40 | 100.0% | 3rd | 5 | 12.5% | -- | Wang XM, et al. Shan Dong Yi Yao. 2009;49:29-30. | Chinese |
| Asia | Eastern Asia | China, Hunan | 2009 | HybriMax (HybriBio Limited, China) | 300 | 297 | 99.0% | 4th | 41 | 12.3% | . | Chen JH, et al. Chin J Mod Med. 2009;25:407-9. | Chinese |
| Asia | Eastern Asia | China, Zhejiang | 2007-2008 | HPV gene chip | 1169 | 1072 | 91.7% | 3rd | 123 | 11.5% | 6.5% | Chen ZB, et al. Chin Gen Prac. 2010;13:1871-3. | Chinese |
| Asia | Eastern Asia | China, Hunan | 2004-2006 | INNO-LiPA | 46 | 44 | 95.7% | 2nd | 5 | 11.4% | -- | Wei Y, et al. J Prac Obstet Gynecol. 2010;26:507-9. | Chinese |
| Asia | Eastern Asia | China, Heilongjiang | 1996-2001 | Restriction fragment length polymorphism | 59 | 53 | 89.8% | 3rd | 6 | 11.3% | -- | Geng XX, et al. Chin Tumor. 2005;14:619-21. | Chinese |
| Asia | Eastern Asia | China, Guangdong | 2007-2008 | HybriMax (HybriBio Limited, China) | 97 | 86 | 88.7% | 3rd | 9 | 10.5% | -- | Peng YP, et al. J Sun Yat-sen Uni. 2011;32:758-63. | Chinese |
| Asia | Eastern Asia | China, Chengdu | 2007-2009 | HPV GenoArray test kit (HybriBio Limited, Hong Kong) | 144 | 116 | 80.6% | 2nd | 12 | 10.3% | 7.1% | Li J, et al. Int J Gynaecol Obstet. 2011;112:131-4. | English |
| Asia | Eastern Asia | Republic of Korea | 2000-2002 | HPV DNA chip | 62 | 59 | 95.2% | 3rd | 6 | 10.2% | -- | Park TC, et al. DNA Cell Biol. 2004;23:119-25. | English |
| Asia | Eastern Asia | China, Zhejiang | 2004-2006 | Restriction fragment length polymorphism | 181 | 172 | 95.0% | 2nd | 17 | 9.9% | -- | Hong D, et al. Int J Gynecol Cancer. 2008;18:104-9. | English |
| Asia | Eastern Asia | China, Hong Kong Special Administration Region | 1997-2007 | INNO-LiPA | NA | 232 | NA | 4th | 23 | 9.9% | -- | Chan PK, et al. Int J Cancer. 2009;125:1671-7. | English |
| Asia | Eastern Asia | Taiwan | 1992-1999 | PCR-ELISA kit (Roche) and sequencing | 152 | 149 | 98.0% | 4th | 14 | 9.4% | 3.6% | Huang LW, et al. J Clin Virol. 2004;29:271-6. | English |
| Asia | Eastern Asia | Japan | 1995-2000 | Restriction fragment length polymorphism | 84 | 64 | 76.2% | 2nd | 6 | 9.4% | -- | Harima Y, et al. Int J Radiat Oncol Biol Phys. 2002;77:1605-6. | English |
| Asia | Eastern Asia | China, Liaoning | 2007-2009 | Microarray hybridization | 154 | 128 | 83.1% | 2nd | 12 | 9.4% | -- | Sun ZR, et al. Int J Gynecol obstet. 2010;109:105-9. | English |
| Asia | Eastern Asia | China, Hong Kong Special Administration Region | 2007^3^ | Sequencing | 96 | 86 | 89.6% | 4th | 8 | 9.3% | -- | Liu SS, et al. Tumor Biol. 2008;29:105-13. | English |
| Asia | Eastern Asia | Republic of Korea | 2003^3^ | HPV-DNA chip (Biomedlab co., Korea) | 72 | 65 | 90.3% | 3rd | 6 | 9.2% | -- | Hwang TS, et al. Gynecol Oncol. 2003;90:51-6. | English |
| Asia | Eastern Asia | China, Shandong | 2007-2010 | HPV GenoArray test kit (HybriBio Limited, Hong Kong) | 198 | 161 | 81.3% | 2nd | 14 | 8.7% | -- | Yuan X, et al. Arch Gynecol Obstet. 2011;283:1385-9. | English |
| Asia | Eastern Asia | China, Liaoning | 2007-2010 | HPV GenoArray test kit (HybriBio Limited, Hong Kong) | 767 | 681 | 88.8% | 2nd | 57 | 8.4% | -- | Wang S, et al. BMC Cancer. 2012;12:160. | English |
| Asia | Eastern Asia | Republic of Korea | 2005^3^ | HPV DNA chip | 53 | 49 | 92.5% | 3rd | 4 | 8.2% | -- | Lee GY, et al. Int J Gynecol Cancer. 2005;15:81-7. | English |
| Asia | Eastern Asia | Republic of Korea | 2007^3^ | HPV DNA chip (MYGene co., Korea) | 133 | 111 | 83.5% | 3rd | 9 | 8.1% | 3.9% | Lee HS, et al. Int J Gynecol Cancer. 2007;17:497-501 | English |
| Asia | Eastern Asia | Japan | 1993-2000 | Sequencing | 356 | 311 | 87.4% | 3rd | 25 | 8.0% | -- | Asato T, et al. J Infect Dis. 2004;189:1829-32. | English |
| Asia | Eastern Asia | China, Huber | 2004-2006 | Sequencing | 112 | 105 | 93.8% | 4th | 8 | 7.6% | 6.3% | Cai HB, et al. Oncology. 2009;76:157-61. | English |
| Asia | Eastern Asia | China, Guangxi | 2010-2011 | HybriMax (HybriBio Limited, China) | 160 | 144 | 90.0% | 3rd | 11 | 7.6% | -- | Ou-Ya M, et al. Chin J of Oncol Prev and Treat. 2012;4:197-9. | Chinese |
| Asia | Eastern Asia | China, Beijing | 2007^3^ | Multiplex hybridization to liquid bead microarray | 102 | 93 | 91.2% | 3rd | 7 | 7.5% | -- | Li Y, et al. Cancer Genet Cytogenet. 2008;182:12-7. | English |
| Asia | Eastern Asia | China, Qinzhou | 2009-2010 | PCR typing diagnostic kit (Kaipu) | 76 | 69 | 90.8% | 2nd | 5 | 7.2% | -- | Yang DQ, et al. Prac Clin Med. 2010;11:113-5. | Chinese |
| Asia | Eastern Asia | China, Inner Mongolian Autonomous Region | 2004-2007 | Type-specific PCR | 77 | 72 | 93.5% | 3rd | 5 | 6.9% | 0.0% | Wu E, et al. Int J Gynecol Cancer. 2009;19:919-23. | English |
| Asia | Eastern Asia | China | 1999-2001 | Sequencing and linear array HPV genotyping (Roche) | 106 | 88 | 76.5% | 3rd | 6 | 6.8% | 5.7% | Liu J, et al. Gynecol Oncol. 2004;94:803-10. | English |
| Asia | Eastern Asia | Taiwan | 1988-1999 | Easychip HPV genotyping array (King Car, Taiwan) | 149 | 149 | 100.0% | 3rd | 10 | 6.7% | -- | Huang HJ, et al. Int J Gynecol Cancer. 2004;14:639-49. | English |
| Asia | Eastern Asia | China, Hubei | 2003-2004 | Sequencing | 110 | 104 | 94.5% | 2nd | 7 | 6.7% | -- | Cai HB, et al. Eur J Gyneacol Oncol. 2008;29:72-5. | English |
| Asia | Eastern Asia | China, Zhengzhou | 2008-2011 | HybriMax (HybriBio Limited, China) | 80 | 76 | 95.0% | 3rd | 5 | 6.6% | -- | Wang Y, et al. Med Innovation of China. 2012;9:83-5. | Chinese |
| Asia | Eastern Asia | Republic of Korea | 1992-1995 | HPV DNA chip | 68 | 52 | 76.5% | 4th | 3 | 5.8% | -- | Lee SA, et al. Cancer Lett. 2003;198:187-92. | English |
| Asia | Eastern Asia | China, Gansu | 2009-2010 | Hybridization | 77 | 76 | 98.7% | 3rd | 4 | 5.3% | -- | Zhou QY, et al. Chin J Healthy Birth & Child Care. 2011;17:5-8. | Chinese |
| Asia | Eastern Asia | India | 2005-2007 | Multiplex PCR/APEX assay | 113 | 104 | 92.0% | 3rd | 5 | 4.8% | 1.80% | Deodhar K, et al. J Med Virol. 2012;84:1054-60. | English |
| Asia | Eastern Asia | China, Zhejiang | 2008-2011 | Hybridization | 103 | 86 | 83.5% | 4th | 4 | 4.7% | 3.0% | Zhu J, et al. PJCCPVD. 2012;20:1833 | Chinese |
| Asia | Eastern Asia | Thailand | 2010-2011 | INNO-LiPA | 155 | 149 | 96.1% | 4th | 9 | 4.7% | 4.00% | Chinchai T, et al. Int J Gynecol Cancer. 2012;22:1063-8. | English |
| Asia | Eastern Asia | China, Yanbian | 1998-2005 | HPV-DNA chip (Biomedlab co., Korea) | 72 | 66 | 91.7% | 4th | 3 | 4.5% | 4.2% | Zhao Y, et al. Pathology Int. 2008;58:643-7. | English |
| Asia | Eastern Asia | Japan | 1995-1999 | Restriction fragment length polymorphism and sequencing | 72 | 67 | 93.1% | 5th | 3 | 4.5% | 4.2% | Sasagawa T, et al. Cancer Epidemiol Biomarkers Prev. 2001;10:45-52. | English |
| Asia | Eastern Asia | China, Fujian | 2008-2009 | Gene chip by HybriMax (HybriBio Limited, China) | 96 | 91 | 94.8% | 7th | 4 | 4.4% | -- | Wu D, et al. Eur J Obstet Gynecol Reprod Biol. 2010;151:86-90. | English |
| Asia | Eastern Asia | Republic of Korea | 1958-2004 | HPV SPF10-LiPA25 | 742 | 674 | 90.8% | 5th | 29 | 4.3% | 3.9% | Oh JK, et al. Asian Pac J Cancer Prev. 2010;11:993-1000. | English |
| Asia | Eastern Asia | China, Gansu | 2007-2008 | HPV GenoArray test (HybriBio Limited, China) | 82 | 70 | 85.4% | 3rd | 3 | 4.3% | 2.5% | Wu X, et al. J Med Virol. 2009;81:693-702. | English |
| Asia | Eastern Asia | Japan | 1993-1997 | Type-specific PCR | 238 | 214 | 89.9% | 4th | 9 | 4.2% | -- | Nagai Y, et al. Am J Clin Oncol. 2001;24:160-6. | English |
| Asia | Eastern Asia | China, Hong Kong Special Administration Region, Shanghai, Guangzhou, Sichuan and Beijing | 1997-1999 | Sequencing and type-specific PCR | NA | 620 | NA | 3rd | 25 | 4.0% | -- | Lo KW, et al. Int J Cancer. 2002;100:327-31. | English |
| Asia | Eastern Asia | China, Jiangsu | 1978-2012 | Hybridization | 62 | 52 | 83.9% | 4th | 2 | 3.8% | -- | Long X, et al. Int J Lab Med. 2012;33:2958-62. | Chinese |
| Asia | Eastern Asia | China, Qinghai | 2005-2008 | HPV gene chip | 284 | 245 | 86.3% | 4th | 8 | 3.3% | 1.4% | Lu JH, et al. J High Altitude Med. 2009;19:12-5. | Chinese |
| Asia | Eastern Asia | China, Xinjiang | 2004-2006 | HybriMax (HybriBio Limited, China) | 350 | 291 | 83.1% | 4th | 9 | 3.1% | -- | Abliz G, et al. J Xinjiang Med Uni. 2009;32:513-7. | Chinese |
| Asia | Eastern Asia | China, Guangxi | 2006-2009 | HPV gene chip | 131 | 129 | 98.5% | 4th | 4 | 3.1% | -- | Ye J, et al. Shandong Med J. 2010;46:20-2. | Chinese |
| Asia | Eastern Asia | China, Huaian | 2007-2010 | HybriMax (HybriBio Limited, China) | 33 | 32 | 97.0% | 3rd | 3 | 3.0% | -- | Zhang JM, et al. J Clin Transfus Lab Med. 2011;`2:117-20. | Chinese |
| Asia | Eastern Asia | China, Macao | 2003-2009 | INNO-LiPA | 36 | 35 | 97.2% | 5th | 1 | 2.9% | 2.8% | Hlaing T, et al. J Med Virol. 2010;82:1600-5. | English |
| Asia | Eastern Asia | China, Liaoning | 2002-2003 | HPV DNA Chip | 45 | 36 | 80.0% | 3rd | 1 | 2.8% | 0.0% | Piao JW, et al. Maternal and Child Health Care of China. 2009;24:662-4. | Chinese |
| Asia | Eastern Asia | China, Guangdong | 2007-2009 | Hybridization | 163 | 154 | 94.5% | 5th | 4 | 2.6% | -- | Peng JX, et al. Int J Lab Med. 2010;31:809-11. | Chinese |
| Asia | Eastern Asia | China, Guangdong | 2009-2010 | HybriMax (HybriBio Limited, China) | NA | 39 | NA | 4th | 1 | 2.6% | -- | Xiao LS, et al. Guangdong Med J. 2010;31:1848-9. | Chinese |
| Asia | Eastern Asia | China, Xinjiang | 2006-2007 | HPV gene chip | 91 | 80 | 87.9% | 3rd | 2 | 2.5% | -- | Abudukadeer A, et al. Eur J Gynecol Oncol. 2010;31:315-8. | English |
| Asia | Eastern Asia | Japan | 2003^3^ | Restriction fragment length polymorphism | 53 | 47 | 88.7% | 5th | 1 | 2.1% | 1.9% | Tsuda H, et al. Gynecol Oncol. 2003;91:476-85. | English |
| Asia | Eastern Asia | Japan | 1990-1993 | Restriction fragment length polymorphism | 66 | 50 | 75.8% | 5th | 1 | 2.0% | -- | Saito J, et al. Gynecol Obstet Invest . 2000;49:190-3. | English |
| Asia | Eastern Asia | China | 2004-2006 | INNO-LiPA | 630 | 615 | 97.6% | 6th | 12 | 2.0% | -- | Chen W, et al. Cancer Causes Control. 2009;20:1705-13. | English |
| Asia | Eastern Asia | China, Xinjiang | 2005-2007 | HPV gene chip | 50 | 50 | 100.0% | 3rd | 1 | 2.0% | 0.0% | Lee L, et al. Xinjiang Med J. 2010;40:5-7. | Chinese |
| Asia | Eastern Asia | China, Shantou | 2006-2008 | HPV gene chip | 125 | 116 | 92.8% | 5th | 2 | 1.7% | -- | Qiu XH, et al. Chin Gen Prac. 2011;14:2360-2. | Chinese |
| Asia | Eastern Asia | China, Inner Mongolia Autonomy Region | 1996-2006 | INNO-LiPA | 63 | 59 | 93.7% | 4th | 1 | 1.7% | -- | Yuan DD, et al. Acta Acad Med Sin. 2008;30:187-90. | Chinese |
| Asia | Eastern Asia | China, Hainan | 2009^3^ | HPV gene chip | 200 | 188 | 94.0% | 5th | 2 | 1.1% | 1.0% | He GL, et al. J Prac Obstet Gynecol. 2009;25:407-9. | Chinese |
| Asia | Eastern Asia | China, Zhejiang | 2009-2010 | Hybridization | 46 | 31 | 67.4% | NA | 0 | 0.0% | -- | Chan C. Maternal and Child Health Care of China. 2011;26:2392-3. | Chinese |
| Asia | Eastern Asia | China, Zhejiang | 2004^3^ | Hybridization | 51 | 38 | 74.5% | NA | 0 | 0.0% | -- | Dong XJ, et al. Zhejiang Clin Med J. 2004;6:532-3. | Chinese |
| Asia | Eastern Asia | China, Guangdong | 2007^3^ | Sequencing | 130 | 107 | 82.3% | NA | 0 | 0.0% | -- | Zhang L, et al. J South Med Univ. 2007;27:399-400. | Chinese |
| Asia | Eastern Asia | China, Guangdong | 2007^3^ | HPV gene chip | 61 | 41 | 67.2% | NA | 0 | 0.0% | 0.0% | Liang CH, et al. Jiangxi J Med Lab Sci. 2007;25:299-301. | Chinese |
| Asia | Eastern Asia | China, Beijing | 2002-2007 | Gene chip | 51 | 48 | 94.1% | NA | 0 | 0.0% | 0.0% | Fan WS, et al. Chin J Nosocomiol. 2009;19:745-7. | Chinese |
| Asia | Eastern Asia | China, Beijing | 2004-2005 | Gene array and type-specific PCR | 93 | 93 | 100.0% | NA | 0 | 0.0% | -- | Li AX, et al. Chin J Exp Clin Virol. 2006;20:49-52. | Chinese |
| Asia | Eastern Asia | China, Zhengzhou | 2004-2006 | INNO-LiPA | 40 | 40 | 100.0% | NA | 0 | 0.0% | -- | Wang XJ, et al. J Int Obstet Gynecol. 2011;38:585-7. | Chinese |
| Asia | South-Eastern Asia | Thailand | 2005^3^ | Type-specific PCR | 90 | 78 | 86.7% | 3rd | 9 | 11.5% | 4.8% | Settheetham-ishida W, et al. Microbiol Immunol. 2005;49:417-21. | English |
| Asia | South-Eastern Asia | Thailand | 2004-2006 | Linear array HPV genotyping (Roche) | 99 | 96 | 97.0% | 4th | 7 | 7.3% | 3.2% | Siriaunkgul S, et al. Gynecol Oncol. 2008;108:555-60. | English |
| Asia | South-Eastern Asia | Thailand | 1990-1993 | Reverse-line blot hybridization | 338 | 322 | 95.3% | 3rd | 10 | 3.1% | 2.7% | Chichareon S, et al. J Natl Cancer Inst. 1998;90:50-7. | English |
| Asia | South-Eastern Asia | Philippines | 1991-1993 | Reverse-line blot hybridization | 323 | 303 | 93.8% | 5th | 9 | 3.0% | 2.8% | Ngelangel C, et al. J Natl Cancer Inst. 1998;90:43-9. | English |
| Asia | South-Eastern Asia | 3 countries | 1989-1992 | PCR and 26HR probes | 98 | 95 | 96.9% | 6th | 2 | 2.1% | -- | Bosch FX, et al. J Natl Cancer Inst. 1995;87:796-802 | English |
| Asia | South-Eastern Asia | Indonesia | 2001-2002 | INNO-LiPA | 45 | 43 | 95.6% | NA | 0 | 0.0% | 0.0% | Schellekens MC, et al. Gynecol Oncol. 2004;93:49-53. | English |
| Asia | Southern Asia | India | 2006^3^ | Reverse-line blot hybridization | 119 | 113 | 95.0% | 3rd | 7 | 6.2% | -- | Peedicayil A, Int J Gynecol Cancer. 2006;16:1591-5. | English |
| Asia | Southern Asia | Sri Lanka | 2006-2007 | INNO-LiPA | 108 | 100 | 92.6% | 4th | 3 | 3.0% | -- | Samarawickrema NA, et al. Int J Gynecol obstet. 2011;115:180-2. | English |
| Asia | Southern Asia | India | 2002-2003 | Linear array HPV genotyping (Roche) | 41 | 36 | 87.8% | 4th | 1 | 2.8% | 0.0% | Sowjanya AP, et al. BMC Infect Dis. 2005;5:116. | English |
| Asia | Southern Asia | India | 2010^3^ | Xcytonscreen HPV | 667 | 614 | 92.1% | 7th | 9 | 1.5% | 0.9% | Pillai RM, et al. Int J Gynecol Cancer. 2010;20:1046-51. | English |
| Asia | Southern Asia | India | 2007-2008 | Sequencing and specific real-time PCR | 278 | 255 | 91.7% | 8th | 3 | 1.2% | 0.0% | Basu P, et al. Asian Pacific J Cancer Prev. 2009;10:27-34. | English |
| Asia | Southern Asia | India | 2005-2007 | Multiplex PCR/APEX assay | 180 | 168 | 93.3% | 7th | 2 | 1.2% | 0.6% | Gheit T, et al. Vaccine. 2009;27:636-9. | English |
| Asia | Southern Asia | India | 1998-1999 | PCR enzyme immunoassy | 179 | 178 | 99.4% | 8th | 2 | 1.1% | 0.9% | Franceschi S, et al. Int J Cancer. 2003;107:127-33. | English |
| Asia | Southern Asia | North India | 2009^3^ | DNA microchip (Xcyton, Bangalore, India) | 110 | 102 | 92.7% | 6th | 1 | 1.0% | 0.9% | Singh A, et al. Int J Gynecol Cancer. 2009;19:1642-8. | English |
| Asia | Southern Asia | India | 2003-2004 | Linear array HPV genotyping (Roche) | 106 | 104 | 98.1% | 6th | 1 | 1.0% | 0.0% | Bhatla N, et al. Int J Gynecol Pathol. 2006;25:398-402. | English |
| Asia | Southern Asia | Pakistan | 2004-2008 | Reverse-line blot hybridization | 91 | 83 | 91.2% | NA | 0 | 0.0% |  | Raza SA, et al. Br J Cancer. 2010;102:1657-60. | English |
| Asia | Southern Asia | Nepal | 2006-2007 | Reverse-line blot hybridization | 61 | 54 | 88.5% | NA | 0 | 0.0% | 0.0% | Sherpa AT, et al. Cancer Causes Control. 2010;21:323-30. | English |
| Asia | Western Asia | Syrian Arab Republic | 2009^3^ | Type-specific PCR | 44 | 42 | 95.5% | 6th | 11 | 26.2% | -- | Darnel AD, et al. Clin Microbiol Infect. 2010;16:262-6. | English |
| Asia | Western Asia | Iran | 2002-2008 | Reverse-line blot hybridization | 45 | 45 | 100.0% | 4th | 2 | 4.4% | -- | Usubutun A, et al. Int J Gynecol Pathol. 2009;28:541-8. | English |
| Asia | Western Asia | Turkey | 1993-2004 | HPV SPF10-LiPA25 | 271 | 232 | 85.6% | NA | 0 | 0.0% | 0.0% | Usubutun A, et al. IntJ Gynecol Pathol. 2009;28:541-8. | English |
| Asia | Western Asia | Saudi Arabia | 2011^3^ | Linear array HPV genotyping test (Roche) | 100 | 89 | 89.0% | NA | 0 | 0.0% | 0.0% | Alsbeih G, et al. Gynecol Oncol. 2011;121:522-6. | English |
| Asia | Across sub-regions | 11 countries | 1949-2009 | HPV SPF10-LiPA25 | 2994 | 2641 | 88.2% | 4th | 102 | 3.9% | -- | de Sanjose S, et al. Lancet Oncol. 2010;11:1048-56 | English |
| Europe | Eastern Europe | Czech Republic | 1993-2005 | Reverse-line blot hybridization and sequencing | 86 | 82 | 95.3% | 6th | 3 | 3.7% | -- | Tachezy R, et al. PLoS One. 2011;6:1-8. | English |
| Europe | Eastern Europe | Czech Republic | 1999^3^ | Dot-blot hybridization and sequencing | 49 | 36 | 73.5% | 4th | 1 | 2.8% | -- | Tachezy R, et al. J Med Virol. 1999;58:378-86. | English |
| Europe | Eastern Europe | Russian Federation | 1988-1994 | Sequencing | 159 | 159 | 100.0% | 5th | 2 | 1.3% | -- | van Muyden RC, et al. Cancer. 1999;85:2011-6. | English |
| Europe | Northern Europe | United Kingdom of Great Britain | 2000-2006 | PCR and 14HR probes | 262 | 255 | 97.3% | 5th | 7 | 2.7% | -- | Powell NG, et al. Int J Cancer. 2011;128:1676-82. | English |
| Europe | Northern Europe | Sweden | 1994-1995 | Single-strand conformation polymorphism | 45 | 43 | 95.6% | 5th | 1 | 2.3% | -- | Zhehbe I, et al. J Pathol. 1997;181:270-5. | English |
| Europe | Northern Europe | United Kingdom of Great Britain, Scotland | 2004^3^ | INNO-LiPA | 370 | 325 | 87.8% | 9th | 1 | 0.3% | 0.3% | Cuschieri K, et al. Br J Cancer. 2010;102:930-2. | English |
| Europe | Northern Europe | United Kingdom of Great Britain | 1999^3^ | Dot-blot hybridization | 43 | 43 | 100.0% | NA | 0 | 0.0% | 0.0% | Giannoudis A, et al. Int J Cancer. 1999;83:66-9. | English |
| Europe | Northern Europe | Sweden | 1999-2001 | Sequencing | NA | 45 | NA | NA | 0 | 0.0% | -- | Andersson S, et al. Cancer Detect Prev. 2005;29:37-41. | English |
| Europe | Northern Europe | Sweden | 1969-1995 | Sequencing | 104 | 80 | 76.9% | NA | 0 | 0.0% | -- | Wallin KL, et al. N Engl J Med. 1999;341:1633-8. | English |
| Europe | Northern Europe | Iceland | 1990-2003 | Multiplex PCR | 140 | 129 | 92.1% | NA | 0 | 0.0% | 0.0% | Sigurdsson K, et al. Int J Cancer. 2007;121:2682-7. | English |
| Europe | Northern Europe | Sweden | 2003-2008 | Luminex (Multimetrix, Germany) | 119 | 111 | 93.3% | NA | 0 | 0.0% | -- | Du J, et al. Acta Oncol. 2011;50:1215-9. | English |
| Europe | Southern Europe | Croatia, Rijeka | 1995-2005 | INNO-LiPA | 54 | 50 | 92.6% | NA | 0 | 0.0% | 0.0% | Hadzisejdic I, et al. Coll Antropol. 2006;30:879-83. | English |
| Europe | Southern Europe | Italy | 1997-1999 | HPV-HS BIO and HPV strip detection kit (AB Analitica, Italy) | 40 | 39 | 97.5% | 3rd | 2 | 5.1% | 5.0% | Del Mistro A, et al. Infect Agent Cancer. 2006;1:9. | English |
| Europe | Southern Europe | Italy, Rome | 2001-2006 | HPV SPF10-LiPA25 | 134 | 121 | 90.3% | 4th | 6 | 5.0% | 3.8% | Mariani L, et al. BMC Cancer. 2010;10:259. | English |
| Europe | Southern Europe | Italy | 2006^3^ | INNO-LiPA | 102 | 92 | 90.2% | 5th | 4 | 4.3% | 3.9% | Ciotti M, et al. Oncol Rep. 2006;15:143-8. | English |
| Europe | Southern Europe | Italy | 1999-2008 | Reverse-line blot hybridization | 157 | 142 | 90.4% | 5th | 5 | 3.5% | -- | Carozzi FM, et al. Cancer Epidemiol Biomarkers Prev. 2010;19:2389-400. | English |
| Europe | Southern Europe | Italy | 1996-2006 | Multiplex PCR kit (Qiagen, Germany) | 268 | 251 | 93.7% | 6th | 6 | 2.4% | 1.1% | Sideri M, et al. Vaccine. 2009;27 Suppl 1:A30-3. | English |
| Europe | Southern Europe | Slovenia | 2004-2006 | Sequencing | 278 | 262 | 94.2% | 7th | 3 | 1.1% | -- | Jancar N, et al. Eur J Obstet Gynecol Reprod Biol. 2009;145:184-8. | English |
| Europe | Southern Europe | Portugal | 2005-2006 | Restriction fragment length polymorphism and sequencing | 44 | 44 | 100.0% | NA | 0 | 0.0% | -- | Nobre RJ, et al. J Med Virol. 2010;82:1024-32. | English |
| Europe | Southern Europe | Italy | 1998-2004 | Sequencing | 65 | 53 | 81.5% | NA | 0 | 0.0% | 0.0% | Tornesello ML, et al. J Med Virol. 2006;78:1663-72. | English |
| Europe | Southern Europe | Greece | 1997^3^ | Restriction fragment length polymorphism | 35 | 34 | 97.1% | NA | 0 | 0.0% | -- | Labropoulou V, et al. Sex Transmis Dis. 1997;24:469-74. | English |
| Europe | Southern Europe | Spain | 2003-2011 | INNO-LiPA and microchip array | 37 | 32 | 86.5% | NA | 0 | 0.0% | -- | Mazarico E, et al. Gynecol Oncol. 2012;125:181-5. | English |
| Europe | Western Europe | France | 1995-2005 | INNO-LiPA | 516 | 501 | 97.1% | 8th | 12 | 2.4% | 0.6% | Pretet JL, et al. Int J Cancer. 2008;122:428-32. | English |
| Europe | Western Europe | France | 1986-1994 | Southern blot hybridization and type-specific PCR | 269 | 223 | 82.9% | 4th | 3 | 1.3% | -- | Lombard I, et al. J Clin Oncol. 1998;16:2613-9. | English |
| Europe | Western Europe | France | 1985-2005 | Linear array HPV genotyping (Roche) | 515 | 494 | 95.9% | 7th | 6 | 1.2% | -- | de Cremoux P, et al. Int J Cancer. 2009;124:778-82. | English |
| Europe | Across sub-regions | 10 countries | 1949-2009 | HPV SPF10-LiPA25 | 2364 | 2058 | 87.1% | 10t | 27 | 1.3% | -- | de Sanjose S, et al. Lancet Oncol. 2010;11:1048-56 | English |
| Europe | Across sub-regions | 3 countries | 1989-1992 | PCR and 26HR probes | 86 | 82 | 95.3% | 6th | 1 | 1.2% | -- | Bosch FX, et al. J Natl Cancer Inst. 1995;87:796-802 | English |
| Oceania | Australia and New Zealand | New Zealand, Auckland | 2000-2006 | Sequencing, type-specific PCR and Linear array HPV genotyping (Roche) | 50 | 50 | 100% | 6th | 1 | 2.0% | 2.0% | Williamson D, et al. Aust N Z J Obstet Gynaecol. 2011;51:67-70. | English |
| Oceania | Australia and New Zealand | Australia | 1999-2002 | Sequencing and linear array HPV genotyping (Roche) | 79 | 71 | 89.9% | 4th | 0 | 1.4% | 0.0% | Liu J, et al. Gynecol Oncol. 2004;94:803-10. | English |
| Oceania | Australia and New Zealand | Australia, Melbourne | 1989-1996 | Linear array HPV genotyping (Roche) | 191 | 166 | 86.9% | 7th | 2 | 1.2% | -- | Stevens MP, et al. Int J Gynecol Cancer. 2006;16:1017-24. | English |
| Oceania | Australia and New Zealand | Australia | 1949-2009 | HPV SPF10-LiPA25 | 179 | 170 | 95.0% | NA | 0 | 0.0% | -- | de Sanjose S, et al. Lancet Oncol. 2010;11:1048-56 | English |
| Oceania | Melanesia | Papua New Guinea | 2006-2009 | PapType High risk HPV Detection and Genotyping Kit (Genera Biosystems, Australia) | 55 | 55 | 100.0% | 4th | 2 | 3.6% | -- | Tabone T, et al. Int J Gynecol Cancer. 2012,117:30-2. | English |
| Oceania | Melanesia | Fiji | 2003-2007 | INNO-LiPA | 152 | 148 | 97.4% | 7th | 2 | 1.4 % | -- | Tabrizi SN, et al. Sex Health. 2011;8:338-42. | English |
|  |  |  |  |  |  |  |  |  |  |  |  |  |  |

^1^ No. of HPV58-positive cases regardless of single- or multiple-type infection / total no. of HPV-positive cases.

^2^ % of cases with HPV58 single-type infection + % of cases with HPV58 multiple-type infection × attribution factor. Attribution factor = no. of cases with HPV58 single-type infection / no. of cases with single-type infection of any HPV type.

^3^ Year of publication.

NA, not applicable.
