# Supplementary material for: Meta-Analysis on Prevalence and Attribution of Human Papillomavirus Types 52 and 58 in Cervical Neoplasia Worldwide
Source: PLoS One. 2014 Sep 17;9(9):e107573. doi: 10.1371/journal.pone.0107573 (PMC4168000; doi:10.1371/journal.pone.0107573)
Supplement: Table S4 — Ranking, relative prevalence and attribution of HPV58 among cervical adenocarcinoma and adenosquamous cell carcinoma reported from 22 studies. (DOCX) [file pone.0107573.s004.docx]

**Table S4. Ranking, relative prevalence and attribution of HPV58 among cervical adenocarcinoma and adenosquamous cell carcinoma reported from 22 studies.**

| **Continent** | **Region** | **City/Country** | **Study period** | **HPV typing method** | **No. of cases examined** | **No. of HPV- positive cases** | **HPV positive rate** | **Ranking of HPV58** | **No of HPV58- positive cases** | **Relative prevalence of HPV58^1^** | **Attribution^2^ of HPV58** | **Reference** | **Reporting language** |
| --- | --- | --- | --- | --- | --- | --- | --- | --- | --- | --- | --- | --- | --- |
| Africa | Eastern Africa | Uganda | 1968-1992 | HPV SPF10-LiPA25 | 35 | 20 | 57.9% | NA | 0 | 0.0% | 0.0% | Odida M, et al. BMC Infect dis. 2008;8:85. | English |
| Americas | Latin America and the Caribbean (South America) | Peru | 1996-1997 | Hybridization | 25 | 23 | 92.0% | NA | 0 | 0.0% | 0.0% | Santos C, et al. Br J Cancer. 2001;85:966-71. | English |
| Americas | Northern America | United States of America, New York | 1980-2007 | INNO-LiPA | 55 | 52 | 94.5% | NA | 0 | 0.0% | 0.0% | Quint KD, et al. Gynecol Oncol. 2010;117:297-301. | English |
| Asia | Eastern Asia | China, Liaoning | 2007-2010 | HPV GenoArray test kit (HybriBio Limited, Hong Kong) | 341 | 294 | 86.2% | 2nd | 26 | 8.8% | -- | Wang S, et al. BMC Cancer. 2012;12:160. | English |
| Asia | Eastern Asia | China, Hong Kong Special Administration Region | 2012^3^ | Linear array HPV genotyping (Roche) | 105 | 93 | 88.6% | 3rd | 6 | 6.50% | 1.00% | Chan PK, et al. Int J Cancer. 2011;131:692-705. | English |
| Asia | Eastern Asia | China, Hong Kong Special Administration Region | 1997-2007 | INNO-LiPA | NA | 48 | NA | 4th | 1 | 2.1% | -- | Chan PK, et al. Int J Cancer. 2009;125:1671-7. | English |
| Asia | Eastern Asia | China, Hong Kong Special Administration Region, Shanghai, Guangzhou, Sichuan and Beijing | 1997-1999 | Sequencing and type-specific PCR | NA | 57 | NA | 4th | 1 | 1.8% | -- | Lo KW, et al. Int J Cancer. 2002;100:327-31. | English |
| Asia | Eastern Asia | China, Yanbian | 1998-2005 | HPV-DNA chip (Biomedlab co., Korea) | 28 | 22 | 78.6% | NA | 0 | 0.0% | 0.0% | Zhao Y, et al. Pathol Int. 2008;58:643-7. | English |
| Asia | Eastern Asia | Japan | 1993-1997 | Type-specific PCR | 54 | 35 | 64.8 | NA | 0 | 0.0% | -- | Nagai Y, et al. Am J Clin Oncol. 2001;24:160-6. | English |
| Asia | Eastern Asia | Republic of Korea | 2007^3^ | HPV-DNA chip (MYGene co., Korea) | 27 | 22 | 81.5% | NA | 0 | 0.0% | 0.0% | Lee HS, et al. Int J Gynecol Cancer. 2007;17:497-501 | English |
| Asia | Eastern Asia | China, Guangxi | 2006-2009 | HPV gene chip | 32 | 26 | 81.3% | NA | 0 | 0.0% | -- | Ye J, et al. Shandong Med J. 2010;46:20-2. | Chinese |
| Asia | Eastern Asia | China, Beijing | 2002-2007 | Gene chip | 26 | 23 | 88.5% | NA | 0 | 0.0% | 0.0% | Fan WS, et al. Chin J Nosocomiol. 2009;19:745-7. | Chinese |
| Asia | Eastern Asia | China, Xinjiang | 2005-2007 | HybriMax (HybriBio Limited, China) | 100 | 61 | 61.0% | NA | 0 | 0.0% | 0.0% | Maimaiti Am et al. J Xinjiang Med Uni. 2009;32:518-21. | Chinese |
| Asia | South-Eastern Asia | Indonesia | 2001-2002 | INNO-LiPA | 29 | 28 | 96.6% | 6th | 1 | 3.4% | 0.0% | Schellekens MC, et al. Gynecol Oncol. 2004;93:49-53. | English |
| Asia | South-Eastern Asia | Thailand | 1990-1993 | Reverse-line Blot Hybridization | 39 | 35 | 89.7% | NA | 0 | 0.0% | 0.0% | Chichareon S, et al. J Natl Cancer Inst. 1998;90:50-7. | English |
| Asia | South-Eastern Asia | Philippines | 1991-1993 | Reverse-line blot hybridization | 33 | 30 | 90.9% | NA | 0 | 0.0% | 0.0% | Ngelangel C, et al. J Natl Cancer Inst. 1998;90:43-9. | English |
| Europe | Northern Europe | Sweden | 1959-2003 | Sequencing and PCR- Single-strand conformation polymorphism | 173 | 117 | 67.6% | NA | 0 | 0.0% | -- | Andersson S, et al. Oncol Rep. 2003;10:175-9. | English |
| Europe | Northern Europe | Ireland | 2009^3^ | Linear array HPV genotyping (Roche) | 40 | 31 | 77.5% | NA | 0 | 0.0% | 0.0% | Houghton O, et al. Histopathol. 2010;57:342-50. | English |
| Europe | Northern Europe | Sweden | 2003-2008 | Luminex (Multimetrix, Germany) | 35 | 32 | 91.4% | NA | 0 | 0.0% | -- | Du J, et al. Acta Oncol. 2011;50:1215-9. | English |
| Europe | Southern Europe | Italy | 1999-2008 | Reverse-line Blot Hybridization | 36 | 35 | 97.2% | 5th | 2 | 5.7% | -- | Carozzi FM, et al. Cancer Epidemiol Biomarkers Prev. 2010;19:2389-400. | English |
| Europe | Sothern Europe | Croatia, Rijeka | 1995-2005 | INNO-LiPA | 40 | 37 | 92.5% | NA | 0 | 0.00% | 0.00% | Hadzisejdic I, et al. Coll Antropol. 2006;30:879-83. | English |
| Europe | Western Europe | France | 1986-1994 | Southern blot hybridization and type-specific PCR | 29 | 23 | 79.3% | NA | 0 | 0.0% | -- | Lombard I, et al. J Clin Oncol. 1998;16:2613-9. | English |

^1^ No. of HPV58-positive cases regardless of single- or multiple-type infection / total no. of HPV-positive cases.

^2^ % of cases with HPV58 single-type infection + % of cases with HPV58 multiple-type infection × attribution factor. Attribution factor = no. of cases with HPV58 single-type infection / no. of cases with single-type infection of any HPV type.

^3^ Year of publication.

NA, not applicable.
