# Supplementary material for: Meta-Analysis on Prevalence and Attribution of Human Papillomavirus Types 52 and 58 in Cervical Neoplasia Worldwide
Source: PLoS One. 2014 Sep 17;9(9):e107573. doi: 10.1371/journal.pone.0107573 (PMC4168000; doi:10.1371/journal.pone.0107573)
Supplement: Table S5 — Ranking, relative prevalence and attribution of HPV52 among cervical intraepithelial neoplasia grade 3 reported from 59 studies. (DOCX) [file pone.0107573.s005.docx]

**Table S5. Ranking, relative prevalence and attribution of HPV52 among cervical intraepithelial neoplasia grade 3 reported from 59 studies.**

| **Continent** | **Region** | **City/Country** | **Study period** | **HPV typing method** | **No of cases examined** | **No. of HPV- positive cases** | **HPV positive rate** | **Ranking of HPV52** | **No of HPV52- positive cases** | **Relative prevalence of HPV52^1^** | **Attribution^2^ of HPV52** | **Reference** | **Reporting language** |
| --- | --- | --- | --- | --- | --- | --- | --- | --- | --- | --- | --- | --- | --- |
| Americas | Latin America and the Caribbean (Central America) | Nicaragua | 2001-2005 | INNO-LiPA and Real-time PCR | 68 | 59 | 86.8% | 3rd | 7 | 11.9% | -- | Hindryckx P, et al. Sex Transm Infect. 2006;82:334-6. | English |
| Americas | Latin America and the Caribbean (Central America) | Costa Rica | 1993-1994 | Dot-blot hybridization | 73 | 68 | 93.2% | 6th | 4 | 5.9% | -- | Herrero R, et al. J Infect Dis. 2005;191:1796-807. | English |
| Americas | Latin America and the Caribbean (Central America) | Honduras | 1993-1995 | Sequencing | 45 | 40 | 88.9% | 4th | 1 | 2.5% | -- | Ferrera A, et al. Int J Cancer. 1999;82:799-803. | English |
| Americas | Latin America and the Caribbean (South America) | Brazil | 2006-2009 | Linear array HPV genotyping (Roche) | 44 | 40 | 90.9% | 4th | 2 | 5.0% | -- | Ribeiro AA, et al. Int J Gynecol Pathol. 2011;30:288-94. | English |
| Americas | Latin America and the Caribbean (South America) | Venezuela | 2001-2011 | INNO-LiPA | 149 | 142 | 95.3% | 4th | 7 | 4.9% | 3.4% | Sanchez-Lander J, et al. Cancer Epidemiol. 2012;36:e284-7. | English |
| Americas | Latin America and the Caribbean (South America) | Brazil | 1996-2000 | Dot-blot hybridization | 102 | 84 | 82.4% | 5th | 2 | 2.40% | 1.00% | Fernandes JV, et al. BMC Res Notes. 2010;3:96. | English |
| Americas | Northern America | United States of America | 1997-2001 | Reverse-line blot hybridization | 608 | 601 | 98.8% | 3rd | 90 | 15.0% | 1.9% | Castle PE, et al. Cancer Epidemiol Biomarkers Prev. 2010;19:1675-81. | English |
| Americas | Northern America | United States of America, Oklahoma | 2007^3^ | Linear array HPV genotyping (Roche) | 64 | 63 | 98.4% | 2nd | 9 | 14.3% | -- | Zuna RE, et al. Mod Pathol. 2007;20:167-74. | English |
| Americas | Northern America | United States of America, Oklahoma | 2003-2007 | Linear array HPV genotyping (Roche) | 305 | 304 | 99.7% | 3rd | 35 | 11.5% | 1.7% | Wentzensen N, et al. Int J Cancer. 2009;125:2151-8. | English |
| Americas | Northern America | United States of America, Mississippi | 1992-2002 | Type-specific PCR | 51 | 49 | 96.1% | 4th | 4 | 8.2% | -- | HU L, et al. Mod Pathol. 2005;18:267-73. | English |
| Americas | Northern America | United States of America, California | 2007-2008 | Dot-blot hybridization | NA | 392 | NA | 5th | 28 | 7.1% | -- | Castle PE, et al. Cancer Epidemiol Biomarkers Prev. 2011;20:946-53. | English |
| Americas | Northern America | United States of America, New Mexico | 1980-1999 | Reverse-line blot hybridization | 1213 | 1178 | 97.1% | 5th | 64 | 5.4% | -- | Wheeler CM, et al. J Natl Cancer Inst. 2009;101:475-87. | English |
| Americas | Northern America | Canada | 1998-2005 | Sequencing and real-time PCR | 176 | 159 | 90.3% | 5th | 5 | 3.1% | -- | Antonishyn NA, et al. Arch Pathol Lab Med. 2008;132:54-60. | English |
| Asia | Eastern Asia | China, Beijing | 2004-2005 | HybriMax (HybriBio Limited, China) | 77 | 74 | 96.1% | 3rd | 19 | 25.7% | -- | Tao PP, et al. Chin J Obstet Gynecol. 2006;41:43-7. | Chinese |
| Asia | Eastern Asia | China, Hong Kong Special Administration Region | 2012^3^ | Linear array HPV genotyping (Roche) | 772 | 650 | 84.2% | 2nd | 167 | 25.70% | 7.20% | Chan PK, et al. Int J Cancer. 2011;131:692-705. | English |
| Asia | Eastern Asia | China, Zhejiang | 2006-2009 | Hybridization | 77 | 62 | 80.5% | 2nd | 13 | 21.0% | -- | Zhou YQ, et al. Chin J Health Lab Tech. 2010;20:2868-70. | Chinese |
| Asia | Eastern Asia | Taiwan | 1999-2001 | HPV blot (King Car, Taiwan) and direct sequencing | 676 | 636 | 94.1% | 3rd | 133 | 20.9% | -- | Chao A, et al. Int J Cancer. 2011;128:653-9. | English |
| Asia | Eastern Asia | China, Wuhan | 2008-2009 | Hybridization | NA | 45 | NA | 3rd | 9 | 20.0% | -- | Liu N, et al. Maternal and Child Health Care of China. 2012;27:4693-5. | Chinese |
| Asia | Eastern Asia | China, Yanbian | 1998-2005 | HPV-DNA chip (Biomedlab co., Korea) | 78 | 45 | 57.7% | 3rd | 8 | 17.8% | 10.3% | Zhao Y, et al. Pathol Int. 2008;58:643-7. | English |
| Asia | Eastern Asia | China, Jiangsu | 2010-2011 | HybriMax (HybriBio Limited, China) | 44 | 40 | 90.9% | 3rd | 7 | 17.5% | -- | Li Y, et al. Int J Lab Med. 2012;33:2261-3. | Chinese |
| Asia | Eastern Asia | China, Chenzhou | 2007-2008 | HPV-DNA chip | NA | 69 | NA | 2nd | 10 | 14.5% | -- | Chen XQ, et al. Guide of Chin Med. 2008;19:8-10. | Chinese |
| Asia | Eastern Asia | China, Zhejiang | 2009-2010 | HPV gene chip | NA | 42 | NA | 2nd | 6 | 14.3% | -- | Yip L, et al. Mod Prac Med. 2011;23:155-156. | Chinese |
| Asia | Eastern Asia | Japan | 1992-1999 | Restriction fragment length polymorphism and dot-blot hybridization | 188 | 179 | 95.2% | 3rd | 23 | 12.8% | 12.2% | Matsukura T, et al. Virology. 2001;283:139-47. | English |
| Asia | Eastern Asia | China, Shenzhen | 2003-2004 | HybriMax (HybriBio Limited, China) | NA | 57 | NA | 3rd | 7 | 12.3% | -- | Wu L, et al. Chin J Clin Obstet Gynecol. 2005;6:346-50. | Chinese |
| Asia | Eastern Asia | China, Huaian | 2007-2010 | HybriMax (HybriBio Limited, China) | 48 | 42 | 87.5% | 3rd | 5 | 11.9% | -- | Zhang JM, et al. J Clin Transfus Lab Med. 2011;2:117-20. | Chinese |
| Asia | Eastern Asia | China, Zhejiang | 2007-2010 | xMAP | 42 | 34 | 81.0% | 3rd | 4 | 11.8% | -- | Dong CL, et al. Chin J Nosocomiol. 2011;21:4858-60. | Chinese |
| Asia | Eastern Asia | China, Shandong | 2007-2010 | HPV GenoArray test (HybriBio Limited, Hong Kong) | 247 | 184 | 74.5% | 2nd | 20 | 10.9% | -- | Yuan X, et al. Arch Gynecol Obstet. 2011;283:1385-9. | English |
| Asia | Eastern Asia | China, Beijing | 2005 | HybriMax (HybriBio Limited, China) | 53 | 52 | 98.1% | 3rd | 5 | 9.6% | -- | Yang Y, et al. Chin J Clin Obstet Gynecol. 2006;7:253-6. | Chinese |
| Asia | Eastern Asia | China, Shenzhen | 2009^3^ | HPV-DNA chip | 48 | 45 | 93.8% | 3rd | 4 | 8.9% | -- | Wang XM, et al. Shan Dong Yi Yao. 2009;49:29-30. | Chinese |
| Asia | Eastern Asia | Western China | 2010-2011 | HPV GenoArray test (HybriBio Limited, China) | 276 | 251 | 90.9% | 3rd | 22 | 8.80% | 3.12% | Li J, et al. J Clin Microbiol. 2012;50:1079-81. | English |
| Asia | Eastern Asia | China, Beijing | 2008^3^ | Multiplex hybridization to liquid bead microarray | 74 | 67 | 90.5% | 4th | 5 | 7.5% | -- | Li Y, et al. Cancer Genet Cytogenet. 2008;182:12-7. | English |
| Asia | Eastern Asia | China, Shenyang | 2008-2009 | HybriMax | 65 | 60 | 92.3% | 3rd | 4 | 6.7% | -- | Wang JH, et al. Chi Gen Prac. 2010;13:3242-4. | Chinese |
| Asia | Eastern Asia | Republic of Korea | 2005^3^ | HPV DNA chip | 114 | 101 | 88.6% | 5th | 6 | 5.9% | -- | Lee GY, et al. Int J Gynecol Cancer. 2005;15:81-7. | English |
| Asia | Eastern Asia | China, Zhejiang | 2006-2008 | HPV gene chip | 91 | 85 | 93.4% | 6th | 5 | 5.9% | -- | Ma JT, et al. Chin J Nat Med. 2010;12:17-20. | Chinese |
| Asia | Eastern Asia | China, Guangxi | 2009^3^ | HPV gene chip | 61 | 57 | 93.4% | 6th | 3 | 5.3% | -- | Chang ZY, et al. J Youjiang Med Coll Nat. 2009;31:10-2. | Chinese |
| Asia | Eastern Asia | China, Xinjiang | 2009-2010 | HybriMax (HybriBio Limited, China) | 43 | 39 | 90.7% | 3rd | 2 | 5.1% | -- | Chen , et al. J Bingtuan Med. 2011;30:1-7. | Chinese |
| Asia | Eastern Asia | China, Shanghai | 2011^3^ | HPV gene chip | 98 | 98 | 100.0% | 5th | 5 | 5.1% | -- | Tao K, et al. Maternal and Child Health Care of China. 2011;26:2342-4. | Chinese |
| Asia | Eastern Asia | Republic of Korea | 2003^3^ | HPV-DNA chip (Biomedlab co., Korea) | 51 | 42 | 82.4% | 5th | 2 | 4.8% | -- | Hwang TS, et al. Gynecol Oncol. 2003;90:51-6. | English |
| Asia | Eastern Asia | China, Guizhou | 2007-2011 | NA | 61 | 60 | 98.4% | 6th | 2 | 3.3% | -- | Zhao S, et al. Maternal and Child Health Care of China. 2012;27:1632-5. | Chinese |
| Asia | Eastern Asia | India | 2005-2007 | Multiplex PCR/APEX assay | 72 | 70 | 97.2% | 5th | 2 | 2.9% | 1.40% | Deodhar K, et al. J Med Virol. 2012;84:1054-60. | English |
| Asia | Eastern Asia | China, Zhengzhou | 2004-2006 | INNO-LiPA | 36 | 34 | 94.4% | 4th | 1 | 2.9% | -- | Wang XJ, et al. J Int Obstet Gynecol. 2011;38:585-7. | Chinese |
| Asia | Eastern Asia | China, Hong Kong Special Administration Region | 1999^3^ | Restriction Fragment Length Polymorphism | 60 | 41 | 68.3% | 4th | 1 | 2.4% | -- | Chan PK, et al. J Med Virol. 1999;59:232-8. | English |
| Asia | Eastern Asia | China, Guangdong | 2007 | Hybridization | NA | 30 | NA | NA | 0 | 0.0% | -- | Jiang X, et al. Chin J Clin Oncol Rehabil. 2009;16:205-7. | Chinese |
| Europe | Eastern Europe | Czech Republic | 1993-2005 | Reverse-line blot hybridization and sequencing | 200 | 188 | 94.0% | 6th | 4 | 2.2% | -- | Tachezy R, et al. PLoS One. 2011;6:1-8. | English |
| Europe | Northern Europe | Denmark | 2004-2005 | INNO-LiPA | 141 | 141 | 100.0% | 2nd | 31 | 22.0% | -- | Kjaer SK, et al. Int J Cancer. 2008;123:1864-70. | English |
| Europe | Northern Europe | Iceland | 1990-2003 | Multiplex PCR | 349 | 336 | 96.3% | 4th | 42 | 12.5% | 4.2% | Sigurdsson K, et al. Int J Cancer. 2007;121:2682-7. | English |
| Europe | Northern Europe | Norway | 1990-1997 | Linear array HPV genotyping (Roche) | 806 | 778 | 96.5% | 7th | 28 | 3.60% | -- | Roberts CC, et al. J Clin Virol. 2006;36:277-82. | English |
| Europe | Northern Europe | Norway | 1999-2001 | RNA-based real-time nucleic acid sequence-based amplification assay | 37 | 34 | 91.9% | NA | 0 | 0.0% | -- | Kraus I, et al. Br J Cancer. 2004;90:1407-13. | English |
| Europe | Southern Europe | Croatia, Rijeka | 1995-2005 | INNO-LiPA | 43 | 40 | 93.0% | 5th | 1 | 2.5% | 0.0% | Hadzisejdic I, et al. Coll Antropol. 2006;30:879-83. | English |
| Europe | Southern Europe | Italy, Brescia | 2005-2006 | Linear array HPV genotyping (Roche) | NA | 36 | NA | 2nd | 9 | 25.0% | -- | Gargiulo F, et al. Virus Res. 2007;125:176-82. | English |
| Europe | Southern Europe | Italy | 2010^3^ | Linear array HPV genotyping (Roche) | NA | 109 | NA | 5th | 11 | 10.1% | -- | [De Francesco MA, et al. Intervirology. 2010;53:417-25.](http://www.ncbi.nlm.nih.gov/pubmed?term=%22De%20Francesco%20MA%22%5BAuthor%5D) | English |
| Europe | Southern Europe | Italy | 2000-2007 | Hybridization | 59 | 58 | 98.3% | 4th | 4 | 6.9% | -- | Costa S, et al. Curr Med Res Opin. 2011;27:569-78. | English |
| Europe | Southern Europe | Slovenia | 2005-2008 | INNO-LiPA | 261 | 253 | 96.9% | 6th | 11 | 4.3% | 2.7% | Kovanda A, et al. Acta Dermatoven APA. 2009:18:47-52. | English |
| Europe | Southern Europe | Italy | 1999-2008 | Reverse-line blot hybridization | 385 | 366 | 95.1% | 6th | 13 | 3.6% | -- | Carozzi FM, et al. Cancer Epidemiol Biomarkers Prev. 2010;19:2389-400. | English |
| Europe | Western Europe | Germany | 2001-2002 | Nested multiplex PCR | 144 | 141 | 97.9% | 2nd | 20 | 14.2% | -- | Sotlar K, et al. J Clin Microbiol. 2004;42:3176-84. | English |
| Europe | Western Europe | France | 1999-2005 | INNO-LiPA | 372 | 368 | 98.9% | 4th | 34 | 9.2% | 2.7% | Pretet JL, et al. Int J Cancer. 2008;122:424-7. | English |
| Europe | Western Europe | Germany | 1996-1997 | PCR enzyme immunoassay | 34 | 33 | 97.1% | NA | 0 | 0.0% | -- | Nindl I, et al. J Clin Pathol. 1999;52:17-22. | English |
| Oceania | Australia and New Zealand | Australia, Melbourne | 1989-1996 | Linear array HPV genotyping (Roche) | 180 | 131 | 72.8% | 4th | 12 | 9.2% | -- | Stevens MP, et al. Int J Gynecol Cancer. 2006;16:1017-24. | English |
| Oceania | Melanesia | Fiji | 2003-2007 | INNO-LiPA Belgium) | 112 | 112 | 100.0% | 3rd | 8 | 7.1% | -- | Tabrizi SN, et al. Sex Health. 2011;8:338-42. | English |

^1^ No. of HPV52-positive cases regardless of single- or multiple-type infection / total no. of HPV-positive cases.

^2^ % of cases with HPV52 single-type infection + % of cases with HPV52 multiple-type infection × attribution factor. Attribution factor = no. of cases with HPV52 single-type infection / no. of cases with single-type infection of any HPV type.

^3^ Year of publication.

NA, not applicable.
