# Supplementary material for: Meta-Analysis on Prevalence and Attribution of Human Papillomavirus Types 52 and 58 in Cervical Neoplasia Worldwide
Source: PLoS One. 2014 Sep 17;9(9):e107573. doi: 10.1371/journal.pone.0107573 (PMC4168000; doi:10.1371/journal.pone.0107573)
Supplement: Table S7 — Ranking, relative prevalence and attribution of HPV52 among cervical intraepithelial neoplasia grade 2 reported from 57 studies. (DOCX) [file pone.0107573.s007.docx]

**Table S7. Ranking, relative prevalence and attribution of HPV52 among cervical intraepithelial neoplasia grade 2 reported from 57 studies.**

| **Continent** | **Region** | **City/Country** | **Study period** | **HPV typing method** | **No of cases examined** | **No. of HPV- positive cases** | **HPV positive rate** | **Ranking of HPV52** | **No of HPV52- positive cases** | **Relative prevalence of HPV52^1^** | **Attribution^2^ of HPV52** | **Reference** | **Reporting language** |
| --- | --- | --- | --- | --- | --- | --- | --- | --- | --- | --- | --- | --- | --- |
| Americas | Latin America and the Caribbean (South America) | Brazil | 2006-2009 | Linear array HPV genotyping (Roche) | 39 | 36 | 92.3% | 2nd | 3 | 8.3% | -- | Ribeiro AA, et al. Int J Gynecol Pathol. 2011;30:288-94. | English |
| Americas | Latin America and the Caribbean (South America) | Venezuela | 2001-2011 | INNO-LiPA | 84 | 78 | 92.9% | 4th | 6 | 7.7% | 6.0% | Sanchez-Lander J, et al. Cancer Epidemiol. 2012;36:e284-7. | English |
| Americas | Northern America | United States of America, Oklahoma | 2007^3^ | Linear array HPV genotyping (Roche) | 58 | 57 | 98.3% | 2nd | 9 | 15.8% | -- | Zuna RE, et al. Mod Pathol. 2007;20:167-74. | English |
| Americas | Northern America | United States of America, Oklahoma | 2003-2007 | Linear array HPV genotyping (Roche) | 427 | 419 | 98.1% | 2nd | 64 | 15.3% | 3.6% | Wentzensen N, et al. Int J Cancer. 2009;125:2151-8. | English |
| Americas | Northern America | United States of America, Mississippi | 1992-2002 | Type-specific PCR | 46 | 42 | 91.3% | 3rd | 4 | 9.5% | -- | HU L, et al. Mod Pathol. 2005;18:267-73. | English |
| Americas | Northern America | Canada | 1998-2005 | Sequencing and real-time PCR | 129 | 112 | 86.8% | 7th | 2 | 1.8% | -- | Antonishyn NA, et al. Arch Pathol Lab Med. 2008;132:54-60. | English |
| Asia | Eastern Asia | China, Hong Kong Special Administration Region | 2012^3^ | Linear array HPV genotyping (Roche) | 805 | 692 | 86.0% | 2nd | 203 | 29.30% | 13.20% | Chan PK, et al. Int J Cancer. 2011;131:692-705. | English |
| Asia | Eastern Asia | China, Wuhan | 2005-2008 | HPV GenoArray test (Hybribio Limited, China) | 44 | 31 | 70.5% | 1st | 9 | 29.0% | 13.3% | Jiang JH, et al. Prog Obstet Gynecol. 2009;18:758-65. | Chinese |
| Asia | Eastern Asia | China, Beijing | 2004-2005 | HybriMax (HybriBio Limited, China) | 77 | 71 | 92.2% | 2nd | 20 | 28.2% | -- | Tao PP, et al. Chin J Obstet Gynecol. 2006;41:43-7. | Chinese |
| Asia | Eastern Asia | China, Beijing | 2010-2011 | HybriMax (HybriBio Limited, China) | 42 | 36 | 85.7% | 3rd | 9 | 25.0% | -- | Wang LQ, et al. Chin J Birth Health Hered. 2012;20:77-9. | Chinese |
| Asia | Eastern Asia | China, Wuhan | 2008-2009 | Hybridization | NA | 61 | NA | 2nd | 15 | 24.6% | -- | Liu N, et al. Maternal and Child Health Care of China. 2012;27:4693-5. | Chinese |
| Asia | Eastern Asia | Taiwan | 1999-2001 | HPV blot (King Car, Taiwan) and direct sequencing | 410 | 359 | 87.6% | 1st | 87 | 24.2% | -- | Chao A, et al. Int J Cancer. 2011;128:653-9. | English |
| Asia | Eastern Asia | Japan | 1995-1996 | Restriction fragment length polymorphism | 67 | 60 | 89.6% | 1st | 14 | 23.3% | 20.9% | Yokoyama M, et al. Cancer Lett. 2003;192:171-9. | English |
| Asia | Eastern Asia | Western China | 2010-2011 | HPV GenoArray test (HybriBio Limited, China) | 181 | 149 | 82.3% | 3rd | 33 | 22.10% | 9.70% | Li J, et al. J Clin Micribiol. 2012;50:1079-81. | English |
| Asia | Eastern Asia | China, Zhejiang | 2006-2009 | Hybridization | 226 | 154 | 68.1% | 2nd | 32 | 20.8% | -- | Zhou YQ, et al. Chin J Health Lab Tech. 2010;20:2868-70. | Chinese |
| Asia | Eastern Asia | China, Zhejiang | 2009-2010 | HPV gene chip | NA | 56 | NA | 1st | 11 | 19.6% | -- | Yip L, et al. Mod Prac Med. 2011;23:155-156. | Chinese |
| Asia | Eastern Asia | China, Guangdong | 2010 | HPV DNA chip | NA | 48 | NA | 2nd | 9 | 18.8% | -- | Zhong XY, et al. Guide of Chin Med. 2011;9:39-40. | Chinese |
| Asia | Eastern Asia | Japan | 1992-1999 | Dot-blot hybridization | 100 | 98 | 98.0% | 3rd | 16 | 16.3% | 16.0% | Matsukura T, et al. Virology. 2001;283:139-47. | English |
| Asia | Eastern Asia | China, Yanbian | 1998-2005 | HPV-DNA chip (Biomedlab co., Korea) | 62 | 32 | 51.6% | 3rd | 5 | 15.6% | 8.1% | Zhao Y, et al. Pathol Int. 2008;58:643-7. | English |
| Asia | Eastern Asia | China, Shenyang | 2008-2009 | HybriMax (HybriBio Limited, China) | 96 | 75 | 78.1% | 3rd | 11 | 14.7% | -- | Wang JH, et al. Chi Gen Prac. 2010;13:3242-4. | Chinese |
| Asia | Eastern Asia | China, Beijing | 2005 | HybriMax (HybriBio Limited, China) | 80 | 76 | 95.0% | 3rd | 11 | 14.5% | -- | Yang Y, et al. Chin J Clin Obstet Gynecol. 2006;7:253-6. | Chinese |
| Asia | Eastern Asia | China, Guangxi | 2007-2009 | HPV gene chip | 60 | 49 | 81.7% | 4th | 7 | 14.3% | -- | Yao J, et al. J Prac Obstet Gynecol. 2011;27:34-8. | Chinese |
| Asia | Eastern Asia | Republic of Korea | 2005^3^ | HPV DNA chip | 42 | 37 | 88.1% | 3rd | 5 | 13.5% | -- | Lee GY, et al. Int J Gynecol Cancer. 2005;15:81-7. | English |
| Asia | Eastern Asia | China, Zhejiang | 2007-2009 | Dot-blot hybridization | NA | 38 | NA | 2nd | 5 | 13.2% | -- | Wang LJ, et al. Disease Surveillance. 2009;24:849-51. | Chinese |
| Asia | Eastern Asia | China, Shenzhen | 2003-2004 | HybriMax (HybriBio Limited, China) | NA | 53 | NA | 3rd | 7 | 13.2% | -- | Wu L, et al. Chin J Clin Obstet Gynecol. 2005;6:346-50. | Chinese |
| Asia | Eastern Asia | China, Zhejiang | 2011^3^ | HPV Gene chip (YanengBio, Shenzhen) | 67 | 61 | 91.0% | 4th | 8 | 13.1% | 1.7% | Xie HY. Chin J Birth Health & Heredity. 2011;19:15-7. | Chinese |
| Asia | Eastern Asia | China, Beijing | 2007^3^ | Multiplex hybridization to liquid bead microarray | 56 | 46 | 82.1% | 3rd | 6 | 13.0% | -- | Li Y, et al. Cancer Genet Cytogenet. 2008;182:12-7. | English |
| Asia | Eastern Asia | China, Shandong | 2007-2010 | HPV GenoArray test kit (HybriBio Limited, Hong Kong) | 271 | 162 | 59.8% | 2nd | 21 | 13.0% | -- | Yuan X, et al. Arch Gynecol Obstet. 2011;283:1385-9. | English |
| Asia | Eastern Asia | China, Shenzhen | 2009^3^ | HPV-DNA chip | 69 | 62 | 89.9% | 3rd | 8 | 12.9% | -- | Wang XM, et al. Shan Dong Yi Yao. 2009;49:29-30. | Chinese |
| Asia | Eastern Asia | China, Jiangsu | 2010-2011 | HybriMax (HybriBio Limited, China) | 75 | 63 | 84.0% | 3rd | 8 | 12.7% | -- | Li Y, et al. Int J Lab Med. 2012;33:2261-3. | Chinese |
| Asia | Eastern Asia | China, Huaian | 2007-2010 | HybriMax (HybriBio Limited, China) | 45 | 32 | 71.1% | 3rd | 3 | 9.4% | -- | Zhang JM, et al. J Clin Transfus Lab Med. 2011;2:117-20. | Chinese |
| Asia | Eastern Asia | China, Zhejiang | 2007-2010 | xMAP | 65 | 44 | 67.7% | 3rd | 4 | 9.1% | -- | Dong CL, et al. Chin J Nosocomiol. 2011;21:4858-60. | Chinese |
| Asia | Eastern Asia | China, Zhejiang | 2006-2008 | HPV gene chip | 72 | 65 | 90.3% | 3rd | 5 | 7.7% | -- | Ma JT, et al. Chin J Nat Med. 2010;12:17-20. | Chinese |
| Asia | Eastern Asia | China, Shanghai | 2011^3^ | HPV gene chip | 67 | 66 | 98.5% | 5th | 5 | 7.6% | -- | Tao K, et al. Maternal and Child Health Care of China. 2011;26:2342-4. | Chinese |
| Asia | Eastern Asia | China, Guangdong | 2007 | Hybridization | NA | 31 | NA | 4th | 2 | 6.5% | -- | Jiang X, et al. Chin J Clin Oncol Rehabil. 2009;16:205-7. | Chinese |
| Asia | Eastern Asia | India | 2005-2007 | Multiplex PCR/APEX assay | 74 | 47 | 63.5% | 3rd | 3 | 6.4% | 1.40% | Deodhar K, et al. J Med Virol. 2012;84:1054-60. | English |
| Asia | Eastern Asia | China, Guizhou | 2007-2011 | NA | 53 | 52 | 98.1% | 5th | 3 | 5.8% | -- | Zhao S, et al. Maternal and Child Health Care of China. 2012;27:1632-5. | Chinese |
| Asia | Eastern Asia | China, Chenzhou | 2007-2008 | HPV DNA chip | NA | 76 | NA | 5th | 3 | 3.9% | -- | Chen XQ, et al. Guide of Chin Med. 2008;19:8-10. | Chinese |
| Asia | Eastern Asia | China, Xinjiang | 2009-2010 | HybriMax (HybriBio Limited, China) | 59 | 52 | 88.1% | 5th | 2 | 3.8% | -- | Chen , et al. J Bingtuan Med. 2011;30:1-7. | Chinese |
| Asia | Eastern Asia | China, Guangxi | 2009^3^ | HPV gene chip | 67 | 55 | 82.1% | 6th | 2 | 3.6% | -- | Chang ZY, et al. J Youjiang Med Coll Nat. 2009;31:10-2. | Chinese |
| Asia | Eastern Asia | China, Shenzhen | 2004-2005 | Hybridization | 52 | 43 | 82.7% | 6th | 1 | 2.3% | -- | Guan T, et al. Maternal and Child Health Care of China. 2006;21:960-3. | Chinese |
| Asia | Eastern Asia | China, Zhejiang | 2008-2010 | HPV gene chip | 80 | 62 | 77.5% | 8th | 1 | 1.6% | -- | Zhao J, et al. Chin J Nosocomiol. 2011;21:2027-9. | Chinese |
| Asia | Eastern Asia | China, Beijing | 2004-2005 | Gene array and type specific PCR | 98 | 98 | 100.0% | NA | 0 | 0.0% | -- | Li AX, et al. Chin J Exp Clin Virol. 2006;20:49-52. | Chinese |
| Asia | Eastern Asia | China, Xian | 2010-2012 | Hybridization | 51 | 47 | 92.2% | NA | 0 | 0.0% | -- | Shan Y, et al. Chin j of Clin Research. 2012;25:456. | Chinese |
| Europe | Eastern Europe | Czech Republic | 1993-2005 | Reverse line blot hybridization and sequencing | 111 | 85 | 76.6% | 5th | 6 | 7.1% | -- | Tachezy R, et al. PLoS One. 2011;6:1-8. | English |
| Europe | Northern Europe | Denmark | 2004-2005 | INNO-LiPA | 36 | 35 | 97.2% | 2nd | 10 | 28.6% | -- | Kjaer SK, et al. Int J Cancer. 2008;123:1864-70. | English |
| Europe | Northern Europe | Norway | 2005-2006 | Linear array HPV genotyping (Roche) | 135 | 133 | 98.5% | 3rd | 23 | 17.3% | 4.0% | Sjoeborg KD, et al. Gynecol Oncol. 2010;118:29-34. | English |
| Europe | Northern Europe | Iceland | 1990-2003 | Multiplex PCR | 92 | 85 | 92.4% | 3rd | 12 | 14.1% | 8.2% | Sigurdsson K, et al. Int J Cancer. 2007;121:2682-7. | English |
| Europe | Northern Europe | Norway | 1990-1997 | Linear array HPV genotyping (Roche) | 110 | 107 | 97.3% | 4th | 6 | 5.6% | -- | Roberts CC, et al. J Clin Virol. 2006;36:277-82. | English |
| Europe | Southern Europe | Italy, Brescia | 2005-2006 | Linear array HPV genotyping (Roche) | NA | 31 | NA | 2nd | 9 | 29.0% | -- | Gargiulo F, et al. Virus Res. 2007;125:176-82. | English |
| Europe | Southern Europe | Italy | 2005-2006 | Linear array HPV genotyping (Roche) | NA | 47 | NA | 5th | 5 | 10.6% | -- | Sandri MT, et al. J Med Virol. 2009;81:271-7. | English |
| Europe | Southern Europe | Italy | 2010^3^ | Linear array HPV genotyping (Roche) | NA | 133 | NA | 8th | 9 | 6.7% | -- | [De Francesco MA, et al. Intervirology. 2010;53:417-25.](http://www.ncbi.nlm.nih.gov/pubmed?term=%22De%20Francesco%20MA%22%5BAuthor%5D) | English |
| Europe | Southern Europe | Italy | 1999-2008 | Reverse-line blot hybridization | 144 | 121 | 84.0% | 5th | 6 | 5.0% | -- | Carozzi FM, et al. Cancer Epidemiol Biomarkers Prev. 2010;19:2389-400. | English |
| Europe | Western Europe | Germany | 2001-2002 | Nested multiplex PCR | 119 | 111 | 93.3% | 5th | 11 | 9.9% | -- | Sotlar K, et al. J Clin Microbiol. 2004;42:3176-84. | English |
| Europe | Western Europe | France | 1999-2005 | INNO-LiPA | 121 | 116 | 95.9% | 4th | 9 | 7.8% | 3.5% | Pretet JL, et al. Int J Cancer. 2008;122:424-7. | English |
| Oceania | Australia and New Zealand | Australia, Melbourne | 1996-2005 | Linear array HPV genotyping (Roche) | 204 | 189 | 92.6% | 2nd | 37 | 19.6% | 5.5% | Stevens MP, et al. J Med Virol. 2009;81:1283-91. | English |
| Oceania | Australia and New Zealand | Australia, Melbourne | 1989-1996 | Linear array HPV genotyping (Roche) | 122 | 78 | 63.9% | 2nd | 11 | 14.1% | -- | Stevens MP, et al. Int J Gynecol Cancer. 2006;16:1017-24. | English |

^1^ No. of HPV52-positive cases regardless of single- or multiple-type infection / total no. of HPV-positive cases.

^2^ % of cases with HPV52 single-type infection + % of cases with HPV52 multiple-type infection × attribution factor. Attribution factor = no. of cases with HPV52 single-type infection / no. of cases with single-type infection of any HPV type.

^3^ Year of publication.

NA, not applicable.
